# Statins exhibit anti-tumor potential by modulating Wnt/β-catenin signaling in colorectal cancer

**DOI:** 10.18632/oncotarget.28755

**Published:** 2025-07-21

**Authors:** Sneha Tripathi, Ekta Gupta, Rutika Naik, Satyajeet Khare, Rafeeq Mir, Siddhesh Kamat, Sanjeev Galande

**Affiliations:** ^1^Laboratory of Chromatin Biology and Epigenetics, Indian Institute of Science Education and Research, Pune 411008, India; ^2^Department of Biology, Indian Institute of Science Education and Research, Pune 411008, India; ^3^Symbiosis School of Biological Sciences (SSBS), Symbiosis International (Deemed University), Lavale, Pune 412115, India; ^4^Centre for Interdisciplinary Research and Innovations, University of Kashmir, Srinagar 190006, India; ^5^Center of Excellence in Epigenetics, Department of Life Sciences, Shiv Nadar Institution of Eminence, Delhi-NCR, India

**Keywords:** colorectal cancer, statins, SATB1, Wnt/β-catenin signaling, tumor-suppressive phenotype

## Abstract

Colorectal cancer remains the second leading cause of cancer-related deaths worldwide, highlighting the urgent need for more effective therapies and a deeper understanding of its molecular basis. Drug repurposing has gained traction as a viable strategy to target dysregulated oncogenic pathways. Statins, commonly prescribed for lowering cholesterol, have recently shown potential anti-cancer effects. In this study, we explore how statin treatment influences lipid metabolism, gene expression, and proteomic profiles in colorectal cancer models. Our findings provide direct evidence that statins selectively modulate key components of the Wnt/β-catenin signaling pathway, a major driver of adenoma formation, including members of the special AT-rich sequence-binding (SATB) protein family. We show that statin treatment downregulates SATB1, a known promoter of tumorigenesis in the context of Wnt activation, while simultaneously upregulating SATB2, which plays an opposing role. This reciprocal regulation shifts cellular phenotypes between epithelial and mesenchymal states in 3D spheroid models. Together, these results highlight the therapeutic potential of statins in colorectal cancer and support their consideration in drug repurposing approaches.

## INTRODUCTION

Colorectal cancer (CRC) is the second most prevalent cause of cancer-related deaths globally, underscoring its severity [1]. Despite extensive research efforts aimed at improving outcomes, low survival rates, frequent relapses, and limited treatment effectiveness among CRC patients underscore the ongoing challenge it poses. The most promising outcomes continue to be associated with early detection and the removal of polyps, followed by chemotherapy. Therefore, there is a growing need for combination treatments that address the early stages of CRC. Researchers are increasingly exploring physiological, genetic and epigenetic factors, as well as environmental factors like diet, to emphasize the importance of chemoprevention. The comprehensive exploration of oncogenic and tumor suppressor pathways in CRC has opened up possibilities for therapeutic targets in its early stages.

Lately, a notable approach in cancer therapeutics involves repurposing drugs that are already approved for treating different disorders. These drugs are chosen because their mechanisms of action are well-characterized, targeting physiological pathways known to be disrupted in tumorigenesis. The selection of a repurposed drug primarily hinges on the connection between the drug’s intended target and the observed trends in tumor progression. For instance, there is evidence indicating that individuals with hypercholesterolemia are at higher risk of developing colorectal cancer [2–4]. Several studies have also suggested a direct correlation between the cholesterol pathway and oncogenic signaling pathways responsible for tumorigenesis [5–8] and metastasis [9, 10]. Moreover, CRC patients with elevated cholesterol levels have been reported to experience liver metastasis [11–13]. The established association between hypercholesterolemia and CRC prognosis positions statins as a promising candidate for repurposing as an anti-cancer drug.

Statin drugs are the most effective treatment for hypercholesterolemia because they inhibit the mevalonate pathway, which is responsible for cholesterol synthesis. Statins function as competitive inhibitors of HMG CoA reductase, an enzyme that catalyzes the rate-limiting crucial step in the mevalonate pathway, effectively halting the cascade at an earlier stage [14]. By doing so, they enable cells to uptake free cholesterol from the bloodstream to fulfill their metabolic requirements, ultimately reducing the blood cholesterol levels. Beyond their pivotal role in managing dyslipidemia, reports have indicated that statins may possess anti-inflammatory properties [15, 16] and can be used to treat coronary heart disease [17]. Some studies have explored their anti-neoplastic effects, suggesting that statins can induce apoptosis in breast cancer by targeting mutant p53 [18]. A few meta-analyses and early patient cohort studies have shown a positive correlation between statin use and reduced risk of developing CRC [19–21]. However, it remains unclear whether this effect is related to or independent of the established mode of action on the mevalonate pathway. Therefore, we collectively examined the lipidome, transcriptome, and proteome profiles in CRC lines upon statin treatment, aiming to establish a mechanistic connection.

Our findings strongly suggest that statins effectively mitigate the progression of colorectal tumor both in cultured CRC cells and in mice. The transcriptome and proteome data generated from statin treated cells indicate the emergence of a tumor-suppressive phenotype in CRC cell lines. Additionally, we observed a specific targeting of the Wnt/β-catenin signaling pathway, which has been shown to play a crucial role in the formation of CRC adenomas [22]. Notably, we report a significant decrease in the protein levels of key components of this pathway, including β-catenin, as well as a global regulator SATB1, following statin treatment suggesting that statin indeed mediates a tumor-suppressive phenotype by targeting aberrant Wnt signaling.

SATB1 functions as a chromatin organizer and has been shown to interact with β-catenin [22]. This interaction creates a feed-forward loop that results in the increased expression of both SATB1 and Wnt target genes [22]. Additionally, multiple studies have shown that elevated SATB1 expression is associated with reduced patient survival in CRC [23–25]. In contrast, the role of SATB2, a homolog of SATB1, has been unclear in the context of CRC. While many reports suggest that SATB2 exerts a tumor suppressive effect [26–30], few studies propose that SATB2 upregulation contributes to tumor progression [31]. Despite these observations, definitive evidence to establish the distinct roles of SATB1 and SATB2 and their correlation with each other in the development of CRC is missing. We therefore aimed to examine the dynamic expression of SATB family proteins, both SATB1 and SATB2, as potential therapeutic targets, specifically in the context of statin treatment.

Our study revealed that statins have an opposing effect on SATB1 and SATB2 proteins in colorectal cancer (CRC). Specifically, we observed a time- and dose-dependent downregulation of SATB1 in response to statin treatment [32]. Here, we further demonstrated that statins effectively reverse the expression patterns of SATB1 and SATB2 in both 2D cell cultures and 3D spheroid model systems, leading to a reduction in tumor burden in *in vivo* experiments. These findings are primarily observed at protein level and can be rescued by the supplementation of mevalonate in cell culture. Our comprehensive approach, which incorporates multi-omics analyses and employs various model systems, significantly contributes to the understanding of the statin-mediated specific targeting of the molecular players of the canonical Wnt pathway.

## RESULTS

### Lipid profile of simvastatin treated cells displays characteristics of a tumor suppressive phenotype

We analyzed the lipid, transcript, and protein profiles in colorectal cancer (CRC) cells treated with statins to gain a comprehensive understanding of the physiological and tumorigenic pathways in parallel. We primarily focused on the lipid profile, given the well-established effect of statins on the cholesterol biosynthesis pathway. Evaluating the cholesterol levels in CRC cell lines following statin treatment (simvastatin, unless otherwise specified) was essential for confirming its mode of action. In addition to cholesterol, our targeted lipidomics analysis also included the assessment of oxysterols, prostaglandins, and free fatty acids (FFAs), all of which are known to play a role in tumor progression [33–37].

**Figure 1:**
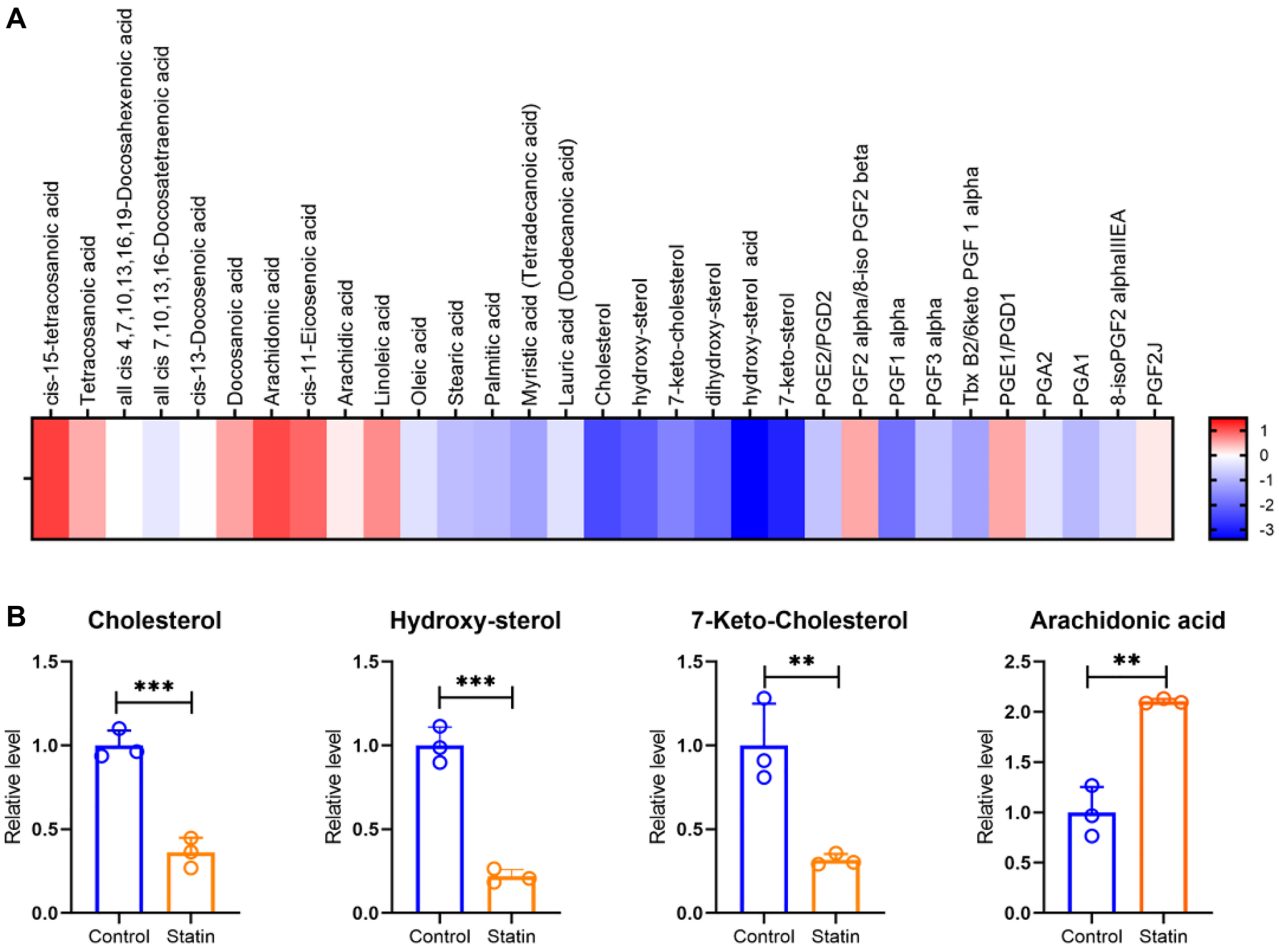
Simvastatin (statin) downregulates cholesterol and its derivatives in CRC cells, validating the canonical mode of action. (**A**) Heatmap to represent the Log 2-fold change levels of free fatty acids (FFAs), cholesterol, oxysterols, and prostaglandins in simvastatin treated CRC line HCT116. No alteration was observed in levels of prostaglandin species and most of the FFAs. However, oxysterols were significantly downregulated. (**B**) Graphs representing the relative levels of cholesterol, hydroxy-sterol, 7-keto-cholesterol, and arachidonic acid on simvastatin treatment as compared to the untreated control set. Significant reduction was observed in cholesterol and its derivatives; however, arachidonic acid was upregulated. Biological replicates *n* = 3, Students *t*-test was performed for statistical significance. ^**^ *p* < 0.001, ^***^ *p* < 0.0001.

Treatment of the CRC cell line HCT116 with simvastatin resulted in a significant reduction in cholesterol levels (Figure 1A). The levels of oxysterols and cholesterol derivatives also exhibited a notable reduction. In addition to oxysterols, prostaglandins are also essential for signaling cascades, as they play a crucial role in relaying information in metabolic pathways [38–41], cell division and differentiation [42]. Therefore, we monitored the levels of prostaglandins upon simvastatin treatment as well, however, we did not observe a significant alteration in the levels of any of the prostaglandins suggesting specificity in the mode of action of simvastatin towards a subset of lipid species as opposed to a global effect (Figure 1A). Further, the levels of most of the free fatty acid (FFA) species remained unaffected, except for stearic acid, palmitic acid, and myristic acid (tetradecanoic acid) which showed a significant decrease upon simvastatin treatment (Figure 1A). Strikingly, we observed a slight increase in arachidonic acid levels, a lipid associated with inflammation (Figure 1B). The inflammatory pathway is regulated by a cascade involving prostaglandins, cytokines, and the NF-κB pathway [43]. Although statin treatment is known to reduce inflammation by downregulating cytokines such as IL-6 and IL-8 [44], our transcriptomics analysis did not reveal alterations in components of the inflammatory pathway. Therefore, the elevated arachidonic acid levels in our lipidomics data might signify a stress response in cells rather than activation of the inflammatory signaling.

Our targeted lipidomics analysis indicated an overall reduction in cholesterol and oxysterol levels, affirming the expected mode of action of statins in CRC cells. While prostaglandins and most FFAs remained unaffected, the observed changes, particularly the reduction in stearic acid, palmitic acid, and myristic acid, suggest a potential tumor-suppressive lipid profile associated with statin treatment. This is noteworthy considering the reported surge and accumulation of lipids in rapidly dividing tumor cells, where cholesterol and FFAs play crucial roles in membrane building, immune response modulation, and drug resistance [8, 45–49]. The dysregulation of FFAs further contributes to tumorigenesis by influencing tumor progression or microenvironment remodeling [50–53]. In summary, the simvastatin mediated reduction in cholesterol may indicate a favorable impact on the lipid profile, aligning with its known anti-tumorigenic effects.

### Transcriptome analysis of simvastatin treated cells reveals effect on multiple tumorigenic pathways including Wnt/β-catenin signaling

**Figure 2:**
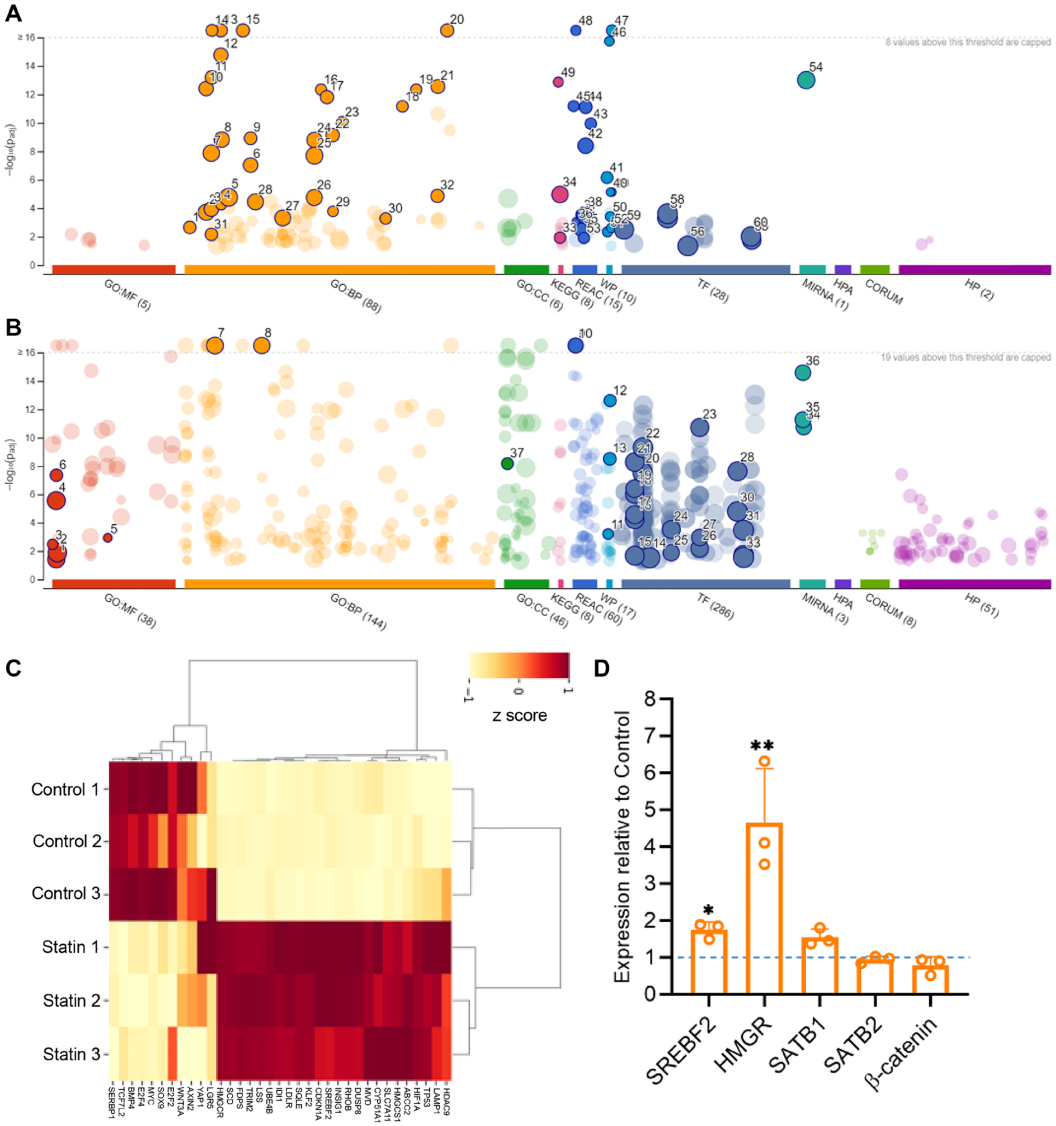
Transcriptome analysis of HCT116 cells upon simvastatin (statin) treatment reveals a tumor-suppressive phenotype. Gene ontology (GO) terms were plotted using g:Profiler and observed separately for upregulated (**A**) and downregulated (**B**) set of genes upon simvastatin treatment of HCT116 cells. The upregulated GO terms specifically involve cholesterol biosynthesis pathway genes, whereas the downregulated terms are related to tumor progressive transcription factors. (**C**) Heatmap for the DE genes significantly expressed in control versus simvastatin-treated sets. The heatmap was generated by selecting a few representative genes of the cholesterol pathway and Wnt target genes using FLASKI online portal (Iqbal, A., Duitama, C., Metge, F., Rosskopp, D., Boucas, J. Flaski. (2021) (https://doi.org/10.5281/zenodo.4849515). (**D**) Quantitation of transcript level alterations in SREBF2, HMGR, SATB1, SATB2 and β-catenin was performed on simvastatin treated cells. Biological replicates *n* = 3, Students’ *t*-test analysis was performed for statistical significance. ^*^ *p* < 0.05, ^**^ *p* < 0.005.

The importance of examining the entire genetic transcript level upon statin treatment lies in its effect on genes responsive to the cholesterol biosynthesis pathway. It is established that a decrease in cholesterol levels leads to the upregulation of genes involved in cholesterol biosynthesis. The key transcription factor, SREBP2, is sensitive to cellular cholesterol levels through mevalonate and oxysterols. When cholesterol levels decrease, SREBP2 activates genes in the mevalonate pathway, which then replenish cholesterol through neogenesis or uptake. In our transcriptome study, we initially focused on the effect of simvastatin treatment on cholesterol responsive genes in the HCT116 CRC cell line. As anticipated, statin mediated reduction in cholesterol resulted in a significant upregulation of mevalonate pathway genes (Figure 2A). Quantitative RT-PCR analysis of HMGR (coding for HMG CoA reductase) and SREBF2 (coding for SREBP2) expression levels supported this observed increase (Figure 2D). Gene ontology (GO) term analysis reported that the other upregulated set of genes (Figure 2A and Supplementary Table 1) were associated with fatty acid synthesis, miR33 activity, omega 9 fatty acid synthesis, and interleukin 2 family signaling. Notably, the predicted upregulation of transcription factors such as KLF15, and AP2 is known to play a tumor-suppressive role [54, 55].

The GO terms for the downregulated genes were separately plotted (Figure 2B and Supplementary Table 2). Transcription factor (TF) activity prediction indicated an overall reduction in tumorigenic factors. A prominent GO term in this set was the gastric cancer network, emphasizing the canonical Wnt pathway. Many of the downregulated transcription factors including c-Myc, p300, E2F1/2, and TCF-1, are known to be regulated by Wnt/β-catenin signaling [56]. We plotted a heatmap to collectively compare the expression profiles of Wnt target genes and cholesterol-responsive genes (Figure 2C). The analysis revealed a distinct downregulation of Wnt-responsive genes including TCF7 (Supplementary Figure 1E) and upregulation of cholesterol-responsive genes upon simvastatin treatment.

Given the central role of Wnt/ β-catenin signaling and its significance in the initiation of colorectal cancer (CRC), we investigated whether statin treatment affected downstream effectors of this pathway via its upstream players. Mutations in APC and β-catenin encoding genes have been shown to predominantly result in aberrant Wnt/ β-catenin signaling, and approximately 75% of CRC patients harbor these mutations [57, 58]. Interestingly, we found no change in the transcript level of β-catenin upon simvastatin treatment (Figure 2D). Next, we validated the expression of SATB1, another crucial upstream factor with known tumorigenic effects [22, 59]. Both transcriptome analysis and quantitative RT-PCR profiling exhibited no alteration in SATB1 and SATB2 transcripts upon simvastatin treatment (Figure 2D).

Taken together, the transcriptome analysis revealed that statin treatment upregulated cholesterol biosynthesis genes due to a feedback mechanism resulting from lowered cholesterol. Conversely, statin-mediated downregulation of oncogenic players targeted Wnt-responsive genes. Intriguingly, major upstream players of the Wnt pathway, including β-catenin, SATB1, and SATB2 did not exhibit changes at the transcript levels.

### Proteome analysis of simvastatin treated HCT15 cells affirms downregulation of Wnt/ β-catenin signaling

**Figure 3:**
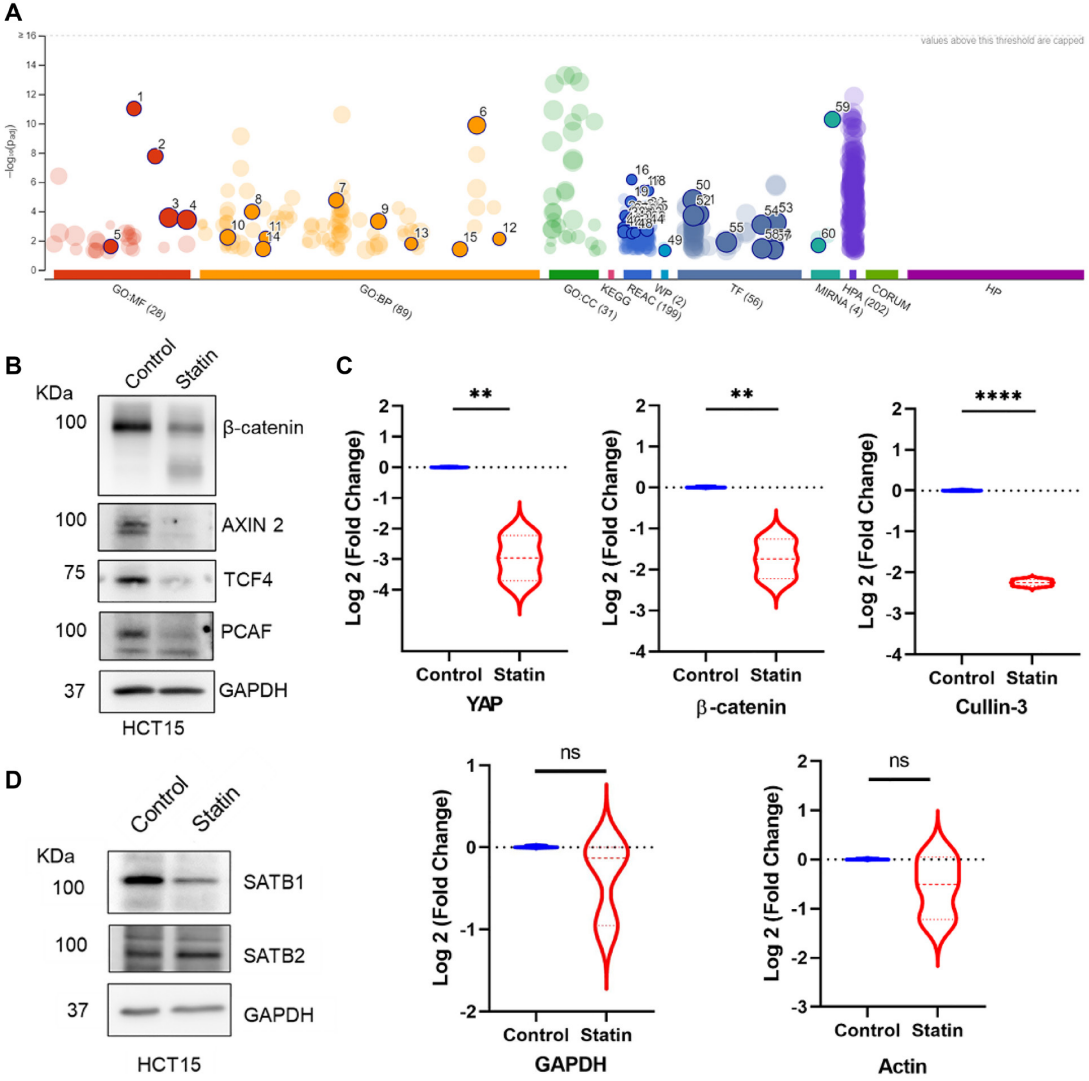
Proteomics analysis depicting significantly downregulated proteins upon simvastatin (statin) treatment reveals Wnt signaling as a major target. (**A**) Downregulated proteins represented in GO term (plotted on g:Profiler) upon statin treatment of HCT15 cells. (**B**) Immunoblot analysis of the effect of simvastatin treatment on select Wnt target proteins revealed their downregulation. (**C**) Log-fold change was observed in the peptide counts of YAP, β-catenin, and Cullin-3 in control versus statin-treated proteomics analysis, whereas no alteration was observed in housekeeping proteins like GAPDH and actin. Biological replicates = 3, with statistical significance of ^**^ *p* < 0.005, ^****^ *p* < 0.00005, ns is non-significant according to Students’ *t*-test analysis. (**D**) Immunoblots depicting SATB1 and SATB2 protein levels in HCT15 cells upon simvastatin treatment. SATB1 protein was significantly reduced, whereas SATB2 expression slightly increased upon simvastatin treatment.

To further confirm the effect of statin treatment, we subjected whole-cell lysate of simvastatin treated HCT15 CRC cells to MS-MS analysis. Remarkably, we observed that simvastatin treatment did not significantly affect the expression levels of most proteins, providing additional evidence for the specificity of mode of action of simvastatin. The commonly categorized off-target effects of the drug, referred to as pleiotropic outcomes, are predominantly linked to its impact on the mevalonate pathway. However, the lack of substantial alterations in the levels of majority of proteins suggests a tightly regulated mechanism mediated by statin. Moreover, there were no subset of proteins that exhibited significant upregulation. The subset of proteins displaying significant alterations were downregulated. Therefore, we performed a GO term analysis for the downregulated proteins to discern the affected pathways (Figure 3A and Supplementary Table 3). The most noteworthy result was the suppression of Wnt/ β-catenin signaling, EGFR signaling, and TGF-β signaling, all of which contribute to tumorigenesis. Additionally, the prediction of reduced transcription factor activity included E2F1/2, p300, and Sp1. We plotted peptide counts for major molecular components such as β-catenin, YAP, and CUL-3 E3 ubiquitin ligase, known for their roles in the Wnt/ β-catenin pathway [60]. The relative expression of these proteins was significantly downregulated upon simvastatin treatment, while the expression of housekeeping genes such as actin and GAPDH remained unchanged (Figure 3C).

We further assessed the effect of simvastatin by monitoring the protein expression levels of key components of the Wnt pathway, including β-catenin, AXIN 2, TCF4, and PCAF. Immunoblot analysis revealed a significant decrease in the levels of all these proteins (Figure 3B and Supplementary Figure 1B, 1F). Thus, simvastatin treatment led to the downregulation of Wnt-responsive genes at both protein and transcript levels. Notably, the upstream regulator β-catenin appeared to be affected only at the protein level, supporting our conclusion that the transcript level changes in the downstream Wnt targets were presumably mediated by alterations in the protein levels of the upstream component. Given the known involvement of SATB1 in Wnt/ β-catenin signaling, we monitored the levels of SATB1 and its homolog SATB2. Immunoblot analysis revealed that SATB1, which is tumorigenic, is downregulated, whereas SATB2 level was not significantly altered upon simvastatin treatment (Figure 3D and Supplementary Figure 1B, 1C, 1G). While a previous report has highlighted the opposing roles of both SATB family proteins [27], their reciprocal expression in various tissues remains less established. Therefore, a comprehensive understanding of the dynamics of the expression profiles of SATB1 and SATB2 is crucial for evaluating them as therapeutic targets of statins. These findings provide insights into the specificity of the anti-tumor mechanism of statins in CRC, particularly targeting the Wnt/ β-catenin signaling (Supplementary Figure 1D). Taken together, the proteomics analysis reinforces the relevance of statin-mediated regulation by specifically targeting tumorigenic players.

### Mevalonate supplementation rescues the effect of simvastatin at protein level

**Figure 4:**
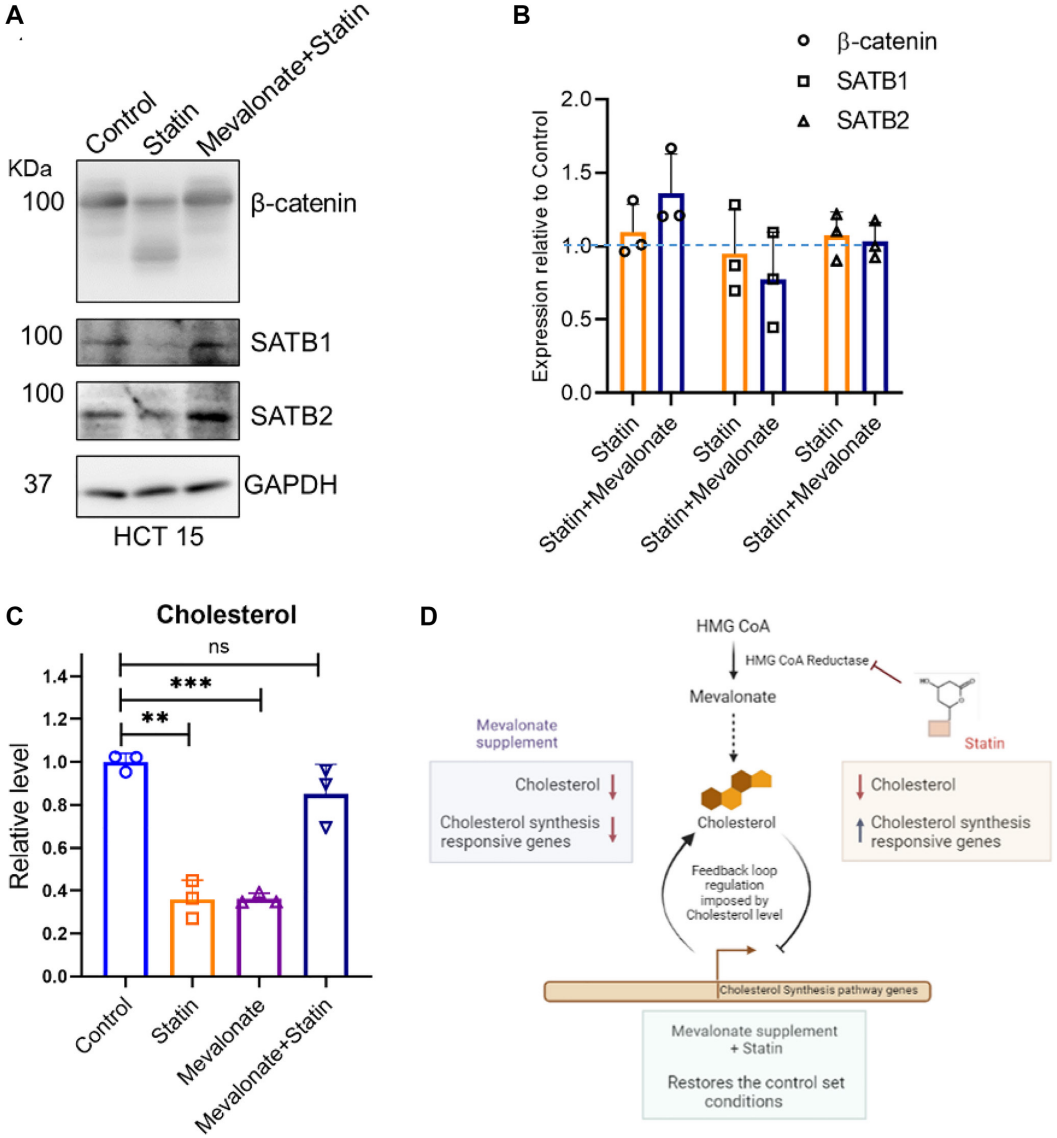
Mevalonate supplementation rescues the effects of simvastatin (statin) on β-catenin and SATB proteins as well as on cholesterol levels. (**A**) Immunoblots depicting protein level alterations in β-catenin, SATB1 and SATB2 upon simvastatin treatment and mevalonate supplementation in HCT15 cells. Mevalonate supplementation resulted in the rescue of the protein levels of β-catenin and SATB1. (**B**) Relative transcript expression of β-catenin, SATB1 and SATB2 upon simvastatin treatment and mevalonate supplementation. No significant changes were observed in the transcript levels of any of these genes. (**C**) Relative levels of cholesterol upon mevalonate supplementation and simvastatin treatment in HCT-15 cells monitored by LC-MS analysis. Cholesterol level was reduced upon treatment with simvastatin or mevalonate and restored upon combined treatment with simvastatin plus mevalonate as compared to the untreated control. Biological replicates *n* = 3, ^**^ *p* < 0.05, ^***^ *p* < 0.005, ns is non-significant as per Students’ *t*-test analysis. (**D**) A model illustrating the impact of these two conditions on the feedback mechanism regulating cholesterol-responsive genes. The decreased cholesterol levels observed with mevalonate supplementation could be attributed to the feedback system, which suppresses cholesterol-responsive gene expression in the presence of excess mevalonate.

Statins act on the mevalonate (MVA) pathway by inhibiting the rate-limiting step catalyzed by HMG CoA reductase. The lactone ring of statin competitively inhibits its substrate, HMG CoA, thus preventing the formation of mevalonate - a precursor essential for cholesterol synthesis [14]. Consequently, we sought to examine the effect of mevalonate supplementation on CRC cell lines when combined with simvastatin treatment. The presence of mevalonate appeared to counteract the simvastatin-induced effects on the upstream components of the Wnt pathway. This was evident as the protein levels of β-catenin and SATB1 were restored, while SATB2 levels remained unchanged (Figure 4A and Supplementary Figures 1H and 2A, 2C, 2D). Consistent with earlier findings, quantitation of the transcript levels of these components did not reveal any significant alterations (Figure 4B and Supplementary Figure 2B). The addition of mevalonate, the product of the enzyme inhibited by statins, facilitated the restoration of the downstream pathway, thereby reversing the effects of simvastatin. Notably, cholesterol levels of the cells treated with mevalonate and simvastatin were comparable to the untreated control group, confirming the rescue in the downstream cascade (Figure 4C). Interestingly, cells supplemented with mevalonate alone exhibited lower cholesterol level compared to the untreated control. This peculiar observation may be explained by considering the feedback mechanism that regulates the transcription of the genes responsible for cholesterol biosynthesis in response to the presence of mevalonate (Figure 4D). In this scenario, the surplus mevalonate presumably led to the downregulation of responsive genes, leading to the downregulation of cholesterol synthesis machinery. We hypothesize that the existing cholesterol may have been depleted by the time of cell harvesting, along with no replenishment from the biosynthetic pathway may have resulted in the observed decreased cholesterol level in the LC-MS readout.

These results underscore the specificity of statin’s effect on CRC, ruling out the possibility of a generic drug response artifact. The restoration of statin-mediated effects upon mevalonate supplementation suggests a regulatory mechanism in Wnt/ β-catenin signaling, potentially linked to the inhibition of the MVA pathway.

### 3D spheroids derived from CRC cell lines exhibit distinct expression pattern of epithelial and mesenchymal markers and Wnt pathway components

**Figure 5:**
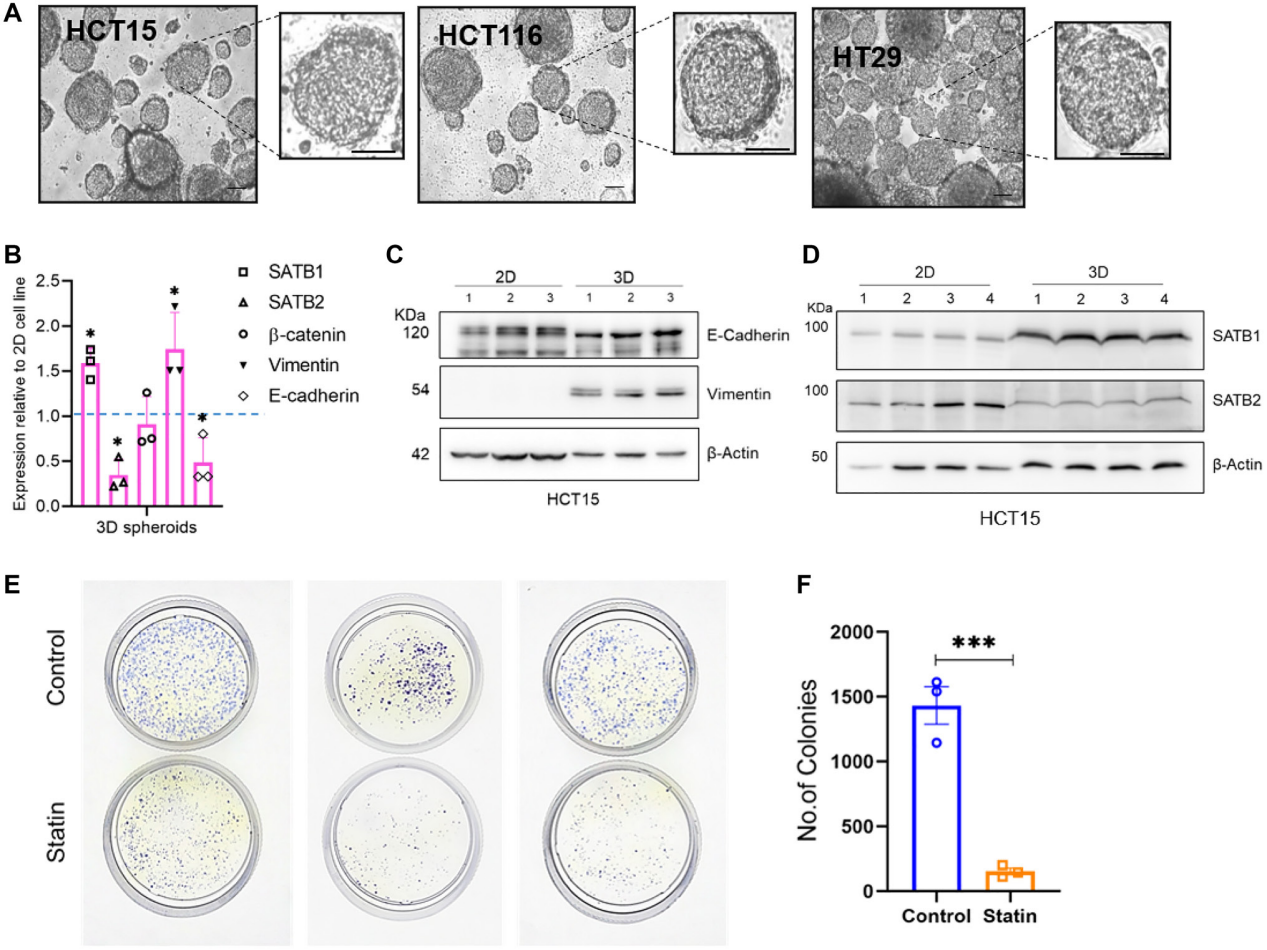
3D spheroids of CRC cells recapitulate the EMT phenotype better than 2D cell cultures. (**A**) Images of spheroids for CRC cell lines HCT15, HCT116, HT29 which were grown in matrigel for 4 days. The panels depict phase contrast images of spheroids, scale bar 50 μm, zoomed in images in adjacent boxes. (**B**) Relative gene expression of SATB1, SATB2, β-catenin and EMT-MET markers, Vimentin and E-cadherin, respectively. The spheroids exhibited a higher expression of EMT marker Vimentin along with a higher expression of SATB1 than SATB2. β-catenin levels were unaltered. (**C**) Immunoblot to validate the protein levels of the EMT marker Vimentin and MET marker E-cadherin in 2D grown HCT15 cells versus the 3D spheroids of HCT15 cells. Vimentin protein is significantly upregulated in 3D spheroids. (**D**) Immunoblot to observe the protein levels of SATB1 and 2 in 2D and 3D spheroids of HCT15 cells recapitulating the tumorigenic phenotype of spheroids. (**E**) Colony formation assay using HCT15 cells upon simvastatin treatment and (**F**) graph representing no. of colonies in control versus treated sets. The colonies were allowed to form on soft agar for 5 days and thereafter treated with simvastatin for additional 48 h. The number of colonies were significantly reduced upon simvastatin treatment. Biological replicates *n* = 3, ^*^ *p* < 0.05, ^**^ *p* < 0.005 as per Students’ *t*-test analysis.

To further confirm the effect of statins on CRC, we opted for a 3-dimensional (3-D) model system than conventional two-dimensional monolayers (2D cell cultures). We established 3D spheroid cultures to investigate the effect of statins on colorectal tumorigenesis (Figure 5A). Spheroid has been a well recognized model system better representing the cellular and molecular transitions during tumorigenesis as compared to monolayer cultures [61]. We initially characterized the spheroids for oncogenic phenotype by assessing the epithelial-to-mesenchymal transition (EMT) and mesenchymal-to-epithelial transition (MET) markers in comparison to 2D cell cultures.

As expected, the 3D spheroids exhibited a more pronounced EMT phenotype, evident from the significantly higher protein and transcript level of the marker Vimentin (Figure 5B, 5C and Supplementary Figure 3A), and lower levels of the MET marker E-cadherin only at transcript level (Figure 5B, 5C and Supplementary Figure 3A). Following the confirmation of the tumorigenic phenotype of spheroids, we monitored the expression of Wnt pathway components β-catenin, SATB1 and SATB2 in both 2D grown cell lines and spheroids. The rationale for investigating the expression profile of Wnt pathway components in a model closer to the tumorigenic phenotype lies in the crucial role played by Wnt/ β-catenin signaling in tumor initiation and progression. It is well established that β-catenin and SATB1 are upregulated in multiple Wnt-driven cancer types including CRC [24]. In our study, the transcript levels of β-catenin showed no significant alteration; however, a dynamic reciprocal expression pattern of SATB proteins was observed, with SATB1 showing higher levels in spheroids (Figure 5B).

In addition to the transcript levels, SATB1 was upregulated at protein level in 3D spheroids compared to 2D cultured cells, while SATB2 levels were downregulated (Figure 5D). This observation is crucial for establishing the dynamics between SATB proteins in the context of tumor progression. Given the established fact that SATB1 is upregulated in tumor tissues [22, 59] and cell lines derived from aggressive adenocarcinomas (Supplementary Figure 5A, 5B), the increased expression of SATB1 in 3D spheroids and the reciprocal expression to SATB2 corroborates the trend.

Notably, we observed an intriguing correlation between the expression of vimentin and the upregulation of SATB1 in 3D spheroids, as well as a connection between E-cadherin and SATB2 in 2D cells. This implies a possible association between the EMT-MET markers and the dynamic expression of SATB proteins. Consequently, it can be inferred SATB1 expression may coincide with the EMT phenotype, while SATB2 might be correlated with the MET phenotype [24]. A study in breast cancer stem cells reported a similar correlation, wherein SATB1 led to the upregulation of Snail1 and Twist1 through the activation of Notch signaling [62]. Conversely, SATB2 has demonstrated variable effects on the generation and regulation of stem cell or progenitor-like cells in CRC [31, 63]. Given that the maintenance of stemness and de-differentiation is a hallmark of tumorigenesis, our inferences in context of both SATB1-SATB2 and EMT-MET phenotypes may contribute to an improved understanding of the role of their dynamic expression in epithelial and mesenchymal transitions.

Having established the 3D model system, next we performed a colony formation assay to assess the impact of statin treatment on the clonogenicity of CRC cells. We observed a significant reduction in the number of colonies upon simvastatin treatment, reaffirming its anti-tumor effect (Figure 5E, 5F). Collectively, these results demonstrate that 3D spheroids derived from CRC cell lines exhibit distinct expression patterns of epithelial and mesenchymal markers and SATB family proteins.

### Simvastatin treated 3D spheroids exhibit reversal of the expression patterns of EMT-MET markers and SATB proteins

**Figure 6:**
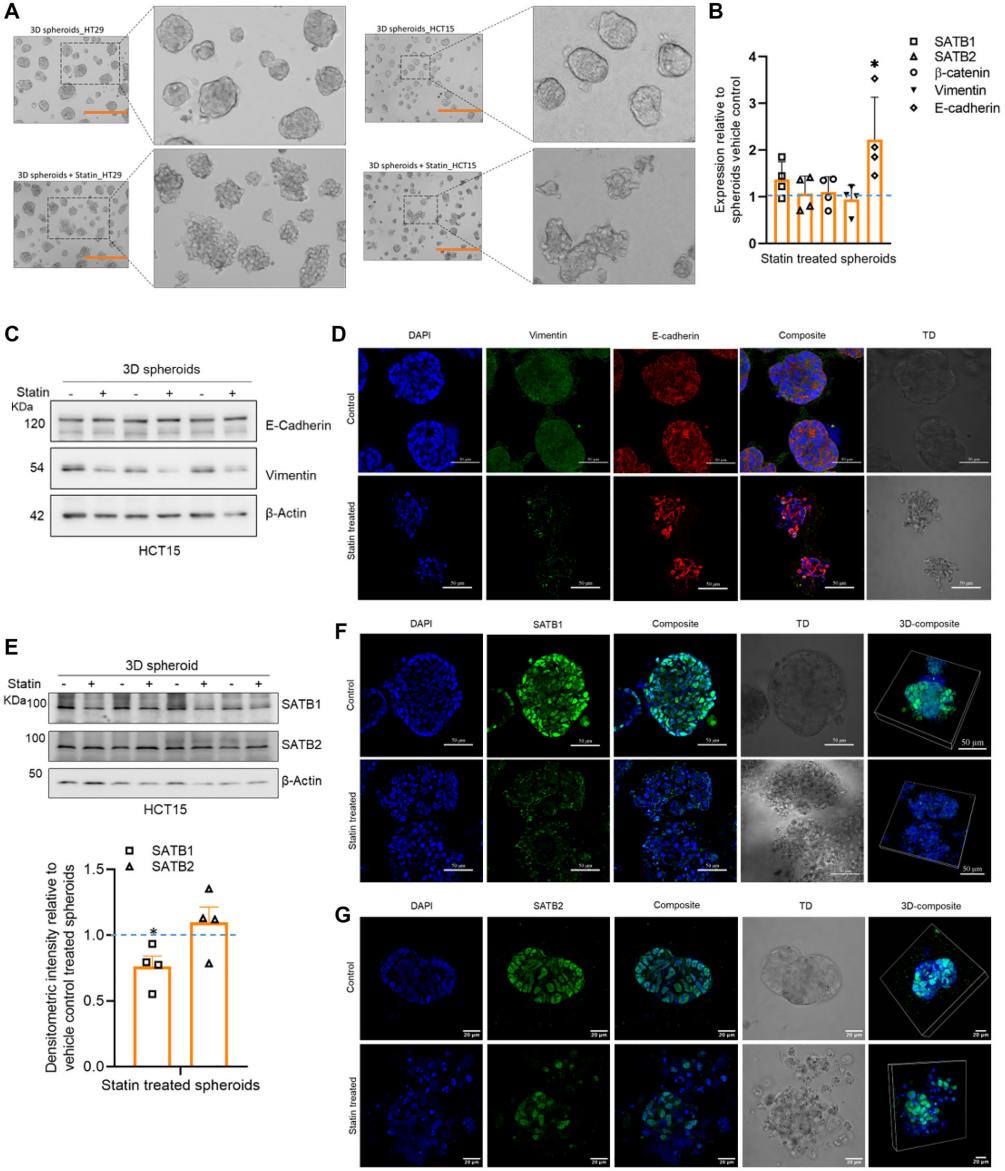
Simvastatin treatment results in downregulation SATB1 protein expression and upregulation of E-cadherin expression in spheroids. (**A**) Images depicting the effect of simvastatin on HT29 and HCT15 spheroids, wherein the treated set depicts disintegration of the spheroids. The images are captured using a phase contrast microscope, scale bar 400 μm (orange). The manually zoomed-in image in the adjacent square box clearly depicts a disintegrated spheroid morphology upon simvastatin treatment. The spheroids were generated over 4 days, thereafter, treated with simvastatin for additional 48 h. (**B**) Relative expression at transcript levels of SATB1, SATB2, β-catenin in spheroids with and without simvastatin treatment recapitulate the results from 2D cell lines of no alteration at transcript level. However, the E-cadherin transcript levels were significantly upregulated upon simvastatin treatment, suggesting transition to an epithelial phenotype. (**C**) Immunoblot to validate the protein expression of EMT marker vimentin and MET marker E-cadherin in untreated and statin treated spheroids respectively. The vimentin protein levels are significantly reduced upon simvastatin treatment. (**D**) Immunofluorescence assay in the 3D spheroids to determine the expression profile of Vimentin and E-cadherin upon statin treatment. The treated spheroids exhibit a disintegrated morphology with significant reduction in vimentin intensity, whereas, E-cadherin remains unaltered, corroborating with the results from immunoblot analysis. Scale bar 50 μm (**E**) Immunoblot to monitor the protein levels of SATB1 and SATB2 in statin-treated spheroids. The densitometry graph below depict the normalized intensities of SATB1 and SATB2. SATB1 levels were significantly reduced, whereas SATB2 levels were not significantly altered upon simvastatin treatment. (**F**) Immunofluorescence assay in the 3D spheroids to determine the expression profile of SATB1 upon simvastatin treatment which is significantly reduced in treated spheroids. The 3D reconstruction panel on the right shows an overall reduction in intensity. Scale bar 50 μm. (**G**) Immunofluorescence assay in the 3D spheroids to determine the expression profile of SATB2 in spheroids. The intensity of SATB2 staining is not altered upon simvastatin treatment, corroborating with the results from immunoblots. The 3D reconstruction of spheroids also depicts a holistic expression of unaltered SATB2 levels. Scale bar 20 μm. Biological replicates *n* = 4, ^*^ *p* < 0.05, ns is non-significant as per Student’s *t*-test analysis.

Next, we treated spheroids derived from the CRC cell lines HT29 and HCT15 with simvastatin and observed their morphology. The treated spheroids exhibited disintegration in both the HT29 and HCT15 cell lines in 3D cultures (Figure 6A). Consequently, the effect of simvastatin on both colonies and 3D spheroids prompted further exploration of transcript and protein alterations, specifically pertaining to the equilibrium in expression levels between the epithelial and mesenchymal markers and the dynamic expression of SATB1 and SATB2 in the spheroid model system. Therefore, we investigated the status of EMT-MET markers in response to simvastatin treatment and observed no significant alterations in vimentin transcript levels. Intriguingly, the epithelial marker E-cadherin exhibited significant upregulation upon simvastatin treatment (Figure 6B). Moreover, immunoblots of extracts from 3D spheroids of the HCT15 cells (Figure 6C) and quantitative immunofluorescence assay (Figure 6D) demonstrated a significant reduction in vimentin at protein level upon simvastatin treatment, suggesting a potential shift in the phenotype from mesenchymal to epithelial following simvastatin treatment (Figure 6C, 6D and Supplementary Figure 3B). An inverse effect was observed on the dynamic expression of SATB proteins as simvastatin treatment led to significant downregulation of SATB1 in both immunoblot (Figure 6E) as well as immunofluorescence assays (Figure 6F), while SATB2 expression remained largely unaltered (Figure 6E, 6G). The transcript levels of both SATB1 and SATB2 remained unaltered in alignment with previous observations (Figure 6B).

In summary, findings from the spheroid experiments suggest a potential link between elevated tumorigenicity (representing a more mesenchymal state) and SATB1 expression, as well as reduced tumorigenicity (reflecting a more epithelial state) and SATB2 expression. Nonetheless, treatment with simvastatin seems to trigger a reversal by altering the expression patterns of both SATB proteins and the markers associated with mesenchymal and epithelial phenotypes, which are typically indicative of tumor advancement.

### Statins reduce tumor burden *in vivo* and recapitulate the effect on SATB proteins

**Figure 7:**
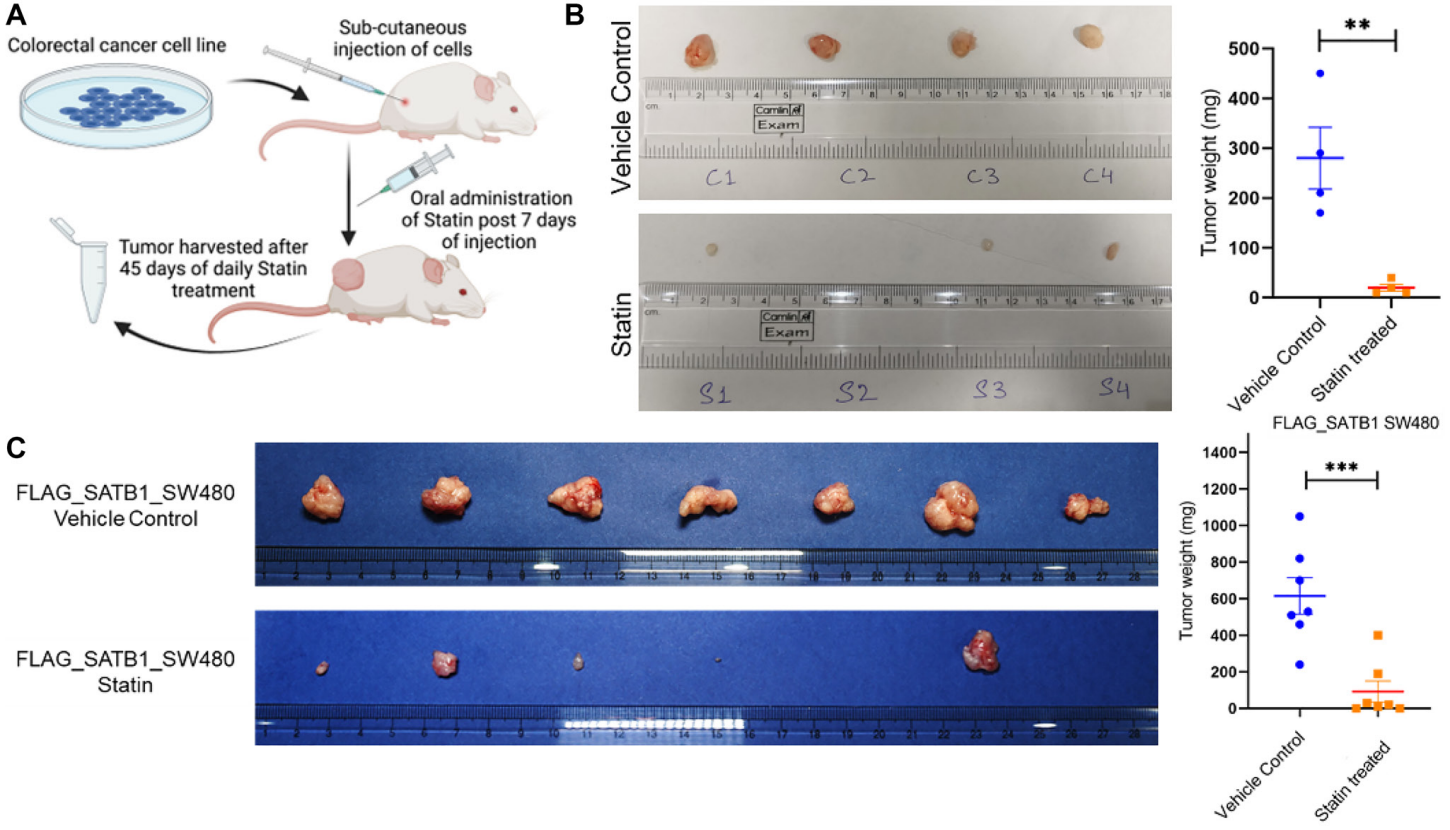
Statin mediated reduction in tumor burden via downregulation of SATB1 *in vivo*. (**A**) Schematic presentation of the experimental strategy using NOD-SCID mice. (**B**) Comparative images of the tumors and graph to show a reduction in tumor burden on simvastatin treatment in mice injected with HCT116 cell line, (Biological replicates *n* = 4) (**C**) FLAG-SATB1 overexpressed SW480 cells were injected sub-cutaneously in NOD-SCID mice, and tumor burden was observed after rosuvastatin treatment (*n* = 7) ^**^ *p* < 0.005, ^***^ *p* < 0.0005 by performing Student’s *t*-test analysis.

We observed a distinct pattern of differential SATB1 and SATB2 expression associated with the EMT-MET phenotype in our spheroid tumorigenic model, which was reversed upon treatment with simvastatin. Given that β-catenin expression remained largely unchanged in both 2D cell lines and their corresponding 3D spheroids, we hypothesized that, in this context, β-catenin may play a less significant role in tumorigenicity compared to the dynamic regulation of SATB proteins. This prompted us to explore the tumorigenic potential of the reciprocal expression profiles of SATB1 and SATB2 *in vivo*. To further validate the anti-tumorigenic potential of statins, we investigated their effects on the dynamic expression patterns of SATB proteins *in vivo*. Using a murine experimental model, CRC cell lines were subcutaneously injected, and tumor burden was monitored over 45 days. Statin treatment was initiated 7 days after the cell injection, administered orally at a dosage of 40 mg/kg/day (Figure 7A). Tumor weights were subsequently measured, and tissue samples were analyzed for transcript and protein expression.

Notably, simvastatin-treated mice exhibited a reduced tumor burden compared to the vehicle control in the mice injected with the aggressive CRC cell line HCT116 (Figure 7B). Considering our hypothesis that SATB1 expression correlates with higher tumorigenicity, we aimed to determine if the molecular effects observed *in vitro* were consistent with statin treatment *in vivo*. To this end, we utilized the less aggressive CRC cell line SW480, modified to stably overexpress FLAG-tagged SATB1 (Supplementary Figure 4A) and administered rosuvastatin to the mice, a statin commonly used for treating patients with high blood cholesterol levels. Following the experimental regimen, rosuvastatin treatment also significantly reduced tumor burden (Figure 7C). Isolated tissues were assessed for SATB1 and SATB2 expression at both transcript and protein levels. While transcript levels remained unchanged, consistent with earlier findings (Supplementary Figure 4B), there was a slight downregulation of SATB1 and a modest upregulation of SATB2 at the protein level (Supplementary Figure 4C).

The unique expression patterns of SATB1 and SATB2 proteins in the tumor samples further support our hypothesis regarding statin-mediated modulation of the canonical Wnt pathway. This highlights SATB proteins as both biomarkers for tumor progression and targets for statin therapy in a Wnt-dependent context. Elucidating the precise mechanisms by which statins influence these key regulators within the Wnt pathway is crucial for advancing the use of this repurposed drug in colorectal cancer therapeutics.

## DISCUSSION

Since its discovery in the 1970s, statins have revolutionized the treatment of hyperlipidemia, marking a significant breakthrough in medical therapeutics [14]. As competitive inhibitors of the rate-limiting step in the mevalonate pathway, statins have transformed approaches to managing conditions such as atherosclerosis, fatty liver disease, and coronary plaques [15, 17]. Their unique lactone ring structure serves as a substrate mimic for HMG-CoA reductase, effectively blocking mevalonate production and, in turn, inhibiting downstream products like cholesterol and its derivatives.

Statins are categorized based on the R group attached to their lactone ring: hydrophobic or hydrophilic [64]. The R group plays a critical role in drug delivery and cellular processivity. Hydrophobic statins, such as simvastatin, fluvastatin, pitavastatin, lovastatin, and atorvastatin, readily penetrate cell membranes. In contrast, hydrophilic statins, including rosuvastatin and pravastatin, become more effective post-metabolism in the liver [64]. These R group variations also influence the pleiotropic side effects seen in earlier formulations [16]. For instance, the first commercially available statin, lovastatin, was associated with side effects like nausea, fatigue, and constipation, which were mitigated in subsequent statins such as simvastatin and pravastatin [65]. Synthetic statins like rosuvastatin and atorvastatin further refined these issues, enhancing efficacy and tolerability [65]. In our study, we utilized both simvastatin and rosuvastatin, and observed comparable effects across *in vitro* and *in vivo* models.

Over the past two decades, interest in the potential anti-cancer properties of statins has grown, driven by their ability to disrupt the cholesterol pathway and deprive tumor cells of essential lipids [66–68]. While earlier studies highlighted the apoptotic and cell cycle-targeting effects of statins on tumorigenesis [69–71], one report indicated specificity in statin effects on APC-mutant CRC cells and patient-derived xenografts [72]. Our investigation centered on the statin-mediated modulation of pathways involved in early adenoma formation in colorectal cancer (CRC). Published data have established a strong link between elevated cholesterol levels and increased Wnt signaling activity [73]. In our transcriptome and proteome analyses, we identified a targeted effect of statins on Wnt-responsive genes. Mechanistically, we observed downregulation of two key Wnt pathway components, β-catenin and SATB1, at the protein level rather than the transcript level in a dose-dependent manner (Supplementary Figure 1A). This suggests that statins influence protein stability, potentially through degradation mechanisms. Our proteomics analysis indicated that statin exerts a specific effect on tumorigenic pathways. Whole-cell lysate proteome profiling revealed no impact of statin on housekeeping proteins such as actin and GAPDH, and the majority of proteins remained unaltered. Notably, the only significant changes were observed in a subset of downregulated proteins, which predominantly included components of oncogenic pathways, particularly targets of Wnt signaling. Quantitative gene expression analysis showed that β-catenin transcript levels remain unaltered, indicating that the effects are restricted to the protein level. This suggests the absence of off-target effects, as such effects would likely result in broader alterations at both transcript and protein levels.

We further hypothesized that the statin-induced modulation of Wnt signaling could be attributed to the inhibition of the mevalonate pathway. This was supported by the restoration of β-catenin and SATB1 protein levels upon mevalonate supplementation. Additionally, our proteomic analysis revealed a decrease in YAP expression, aligning with recent studies that demonstrated YAP downregulation in response to statin treatment via cholesterol pathway inhibition [74–76]. Through this comprehensive analysis, we not only validated established molecular targets of statins but also identified novel targets, such as the SATB family of proteins, which play significant roles in Wnt/β-catenin signaling.

The impact of statins on the SATB family of chromatin organizers is particularly noteworthy, given their crucial role in regulating the expression of downstream Wnt target genes. Previous studies have shown that SATB1 contributes to the upregulation of Wnt-responsive genes in cooperation with β-catenin and TCF/LEF transcription factors [22, 77]. The observed reduction in SATB1 protein levels following statin treatment suggests a direct association with decreased tumorigenicity. Conversely, SATB2, known for its reciprocal expression profile, functions as a tumor suppressor [26]. Although the effect of statins on SATB2 expression was less pronounced compared to SATB1, the dynamic expression patterns of both proteins were disrupted in tumor tissues and 3D spheroids. Furthermore, survival analyses of patients across various cancer types revealed that the expression of SATB family proteins, particularly SATB1 and SATB2, correlates with tissue-specific patterns and may significantly influence disease prognosis [24].

3D spheroids proved to be a superior model system compared to 2D cell lines for investigating the reciprocal expression of SATB1 and SATB2. This was validated through expression profiles of EMT-MET markers, which indicated enhanced tumorigenic potential. Notably, higher SATB1 levels strongly correlated with increased Vimentin expression in spheroids, while SATB2 levels were associated with E-cadherin expression in the corresponding 2D cells. Simvastatin treatment of spheroids not only reversed the SATB protein expression profiles but also led to an increase in E-cadherin and a significant reduction in Vimentin transcript levels, suggesting a shift toward an epithelial phenotype. Morphological observations revealed disintegration of spheroids following statin treatment, indicative of a phenotype transition. These findings highlight an intricate interplay among SATB protein expression, the EMT-MET phenotype, and the tumorigenic versus anti-tumorigenic effects of statins, suggesting a potential regulatory mechanism. Exploring the interplay between SATB chromatin organizers and EMT-MET transitions, both in the presence and absence of statin treatment, presents an exciting opportunity to further investigate the connection between chromatin modifications and cell phenotype. This could reinforce the role of SATB proteins as potential EMT-MET markers associated with tumor progression [24].

The *in vivo* analysis performed using mice further validated the statin mediated effects on SATB proteins. NOD-SCID mice subcutaneously injected with HCT116 cells and SW480 FLAG-SATB1 overexpressing cells exhibited a significant reduction in tumor burden following both simvastatin and rosuvastatin treatment respectively. This reduction was accompanied by decreased SATB1 expression, aligning with the in cellulo observations. We recognize that drawing a direct correlation between in cellulo and *in vivo* doses is inherently complex. *In vivo*, the drug undergoes hepatic metabolism, variable tissue distribution, and interaction with serum components, all of which can significantly lower the concentration of the bioavailable compound compared to in cellulo conditions. Moreover, *in vivo* statin treatment typically extends over several days, whereas the in cellulo experiments, for example, involve 48-hour treatments. For an *in vivo* dose of 40 mg/kg simvastatin, cell-based studies have reported a corresponding minimal effective concentration of approximately 10 μM [78]. Notably, the same study characterized any non-cholesterol-lowering effects of statins as pleiotropic. It is therefore important to differentiate between non-specific off-target effects and specific alternative functions that may be exploited for drug repurposing. Supporting this, our *in vivo* mouse studies reinforce the anti-neoplastic potential of statins, as they show reduced tumor burden without any observable adverse effects [79].

Identifying molecular markers for aggressive diseases remains a considerable challenge despite numerous reported candidates. However, SATB proteins demonstrated consistent expression patterns across both *in vitro* and *in vivo* models. Furthermore, the pronounced impact of statins on the Wnt pathway, with SATB proteins serving as a readout, underscores the importance of these findings. Thus, this study contributes to the growing list of molecular targets for prognosis and treatment, advancing our understanding of the repurposed role of statins in colorectal cancer therapeutics.

## MATERIALS AND METHODS

### Cell culture and spheroid formation

HCT116 and HCT15 CRC cell lines were obtained from the European Collection of Cell Cultures (ECACC) and HT29 cell line was obtained from American Type Culture Collection (ATCC, Manassas, VA, USA). SW480 cell line was obtained from American Type Culture Collection (ATCC, Manassas, VA, USA) and the FLAG-tagged SATB1 over-expression SW480 and empty vector FLAG control stable cell lines were established as described [59]. HCT116 was cultured in DMEM media (Gibco, Waltham, MA, USA) with 10% FBS whereas HCT15 and HT29 were cultured in RPMI media (Gibco, Grand Island, NY, USA) with 10% FBS. Spheroid formation was performed according to the protocol, as described [80]. Briefly, the 24-well dish was coated with matrigel bed and allowed to solidify for 30 min at 37°C. Single-cell suspension of the cells was prepared, and 10^4^ cells were seeded per well. On-top matrigel was added in media and spheroids were allowed to form for 4 days. Matrigel was obtained from Corning (Cat. No. 356231). Simvastatin and rosuvastatin used in all assays were obtained from Sigma (simvastatin Cat No. 79902-63-9, rosuvastatin #SML-1264) at final concentration of 10 μM. Rosuvastatin (Rosulip) used for *in vivo* assays was obtained from Cipla.

### Colony formation assay

Colony formation assay was also performed as described [81]. Briefly, 1.2% low melting point agarose was used as the base with the media. 1 × 10^4^ cells were seeded in a 60 mm dish and topped with 0.6% agarose + media. The colonies were allowed to grow for 7 days in total. The colonies were stained with crystal violet (0.2% final concentration in methanol) for 15 min at RT. The colonies were given thorough wash with PBS to remove extra stain and air-dried before imaging.

### Western blotting and RNA isolation

The antibodies used were as follows, β-catenin (BD Biosciences Cat No. 610153), SATB1_L745 (Cell Signaling Tech, Cat No. 3650), SATB2 (Abcam, Cat No. Ab92447), GAPDH (Abcam, Cat No. G041), TCF4 (CST #2569S), AXIN2 (CST #76G6), PCAF (Santa Cruz #13124), β-Actin (Sigma #A2228), E-cadherin (Abcam #ab40772), Vimentin (Abcam #ab92547). The lysates were prepared in RIPA buffer (20 mM Tris pH7.5, 150 mM NaCl, 1 mM EDTA, 1 mM EGTA, 1% NP-40, 1% Sodium deoxycholate, protease inhibitor cocktail (PIC, Thermo Fisher Scientific Cat No. A32963). Total RNA was extracted from cells grown under 2D and 3D culture conditions and vehicle control and statin-treated sets using Trizol reagent (Taraka, Cat No. 9109). Extracted RNA was either subjected to library preparation for high-throughput sequencing or PCR-based gene expression analysis. For qPCR-based gene expression analysis, cDNA was synthesized using High-capacity cDNA Reverse Transcription kit (Applied Biosystems Cat No. 4368814). The cDNA was utilized for relative gene expression analysis using gene-specific primers, Supplementary Table 4, with 18srRNA as endogenous control.

### TOP-FOP dual-luciferase reporter assay

The TOP and FOP reporter constructs were kindly provided by Dr. R.T. Moon. The TOP construct contains an intact TCF7 binding site, while the FOP construct has a mutated TCF7 binding site. Briefly, HCT116 cells were transfected with the TOP and FOP constructs, along with the Renilla Luciferase reporter construct. Simvastatin treatment was applied 24 hours after transfection, and 48 hours post-treatment, the cell lysates were collected for the assay. Following the kit protocol for the Renilla-Firefly Luciferase Dual Assay Kit (Thermo Scientific, Cat No. 16185), luciferase reagent was added, and bioluminescence was measured. Appropriate negative controls were included, and the experiment was performed in biological triplicates.

### Transcriptome analysis sample preparation

Total RNA (500 ng) was subjected to mRNA purification using NEBNext Poly(A) mRNA Magnetic Isolation Module (NEB, US) according to the manufacturer’s instructions. The purified mRNA was used for library preparation using NEBNext Ultra II RNA Library Prep Kit for Illumina (New England Biolabs, USA) using the protocol provided in the kit. The final libraries were purified using HighPrep PCR Clean-up System (MagBio Genomics, USA) and were quantified using the Qubit 1X HS DNA system (Thermo Fisher Scientific). All libraries were pooled in equimolar ratios and subjected to 75 bp PE sequencing chemistry on Nextseq550, Illumina [82].

### RNAseq analysis

Paired-end sequencing was performed using RNA samples on Illumina platform (Macrogen Inc, Korea). In brief, after performing quality control (QC), qualified samples were processed for library construction. Sequencing library was prepared by random fragmentation of cDNA followed by adapter ligation. Adapter ligated fragments were PCR amplified, and gel purified. The libraries were loaded into a flow cell and each fragment was clonally amplified through bridge amplification [83]. The sequencing data was converted into raw data for analysis. The files have been submitted to SRA database and the accession number for the same is PRJNA957223.

The quality of sequencing reads was checked using FASTQC (version 0.10.1) (Andrews, S. (2010). FastQC: a quality control tool for high throughput sequence data) Available online at: (http://www.bioinformatics.babraham.ac.uk/projects/fastqc/). The sequence alignment was performed on the human genome (version hg38) (https://www.gencodegenes.org/) using HiSAT2 (v 2.05) (https://daehwankimlab.github.io/hisat2/manual/). The resulting bam files were used to generate a count matrix [84] followed by differential expression analysis (v 1.18.1) [85]. Ontology analysis was carried out using clusterProfiler (v 3.6.0) [86, 87]. The color of the circles indicates the specific dataset the terms belong to on the x axis, for example, Red = Molecular Function, Orange = Biological pathway, REAC = Reactome database, TF = Transcription Factor. The size of each circle represents the number of genes associated with that term; larger circles indicate a greater number of genes linked to the term. The higher the circles are positioned on the y-axis, the more significant the term is. The numbered circles highlighted have been chosen to emphasize the major terms that show significant changes, as detailed in the list provided in Supplementary Tables 1–3.

### Lipidomics analysis

All the samples for lipidomics analysis were prepared and analyzed using established protocols previously described by us [88–90] on a Sciex X500R QTOF mass spectrometer, fitted with an Exion series UHPLC. Briefly, the CRC cell line HCT116 was treated with statin and cells were harvested for lipid extraction. Chloroform and methanol (2:1) were used to process the samples. The lipids were run in negative and positive modes in the LC-MS method, keeping Free Fatty Acid (FFA 17:1, 1 nanomole final concentration) as the internal standard.

### Proteomics analysis

Whole cell lysates were resolved on a 10% SDS-PAGE gel and subsequently processed for proteomics using standard in-gel sample preparation protocols [91]. Briefly, the gel bands of interest were excised, destained, reductively alkylated and digested using MS-grade trypsin. Subsequently, the peptides were desalted and processed for proteomics analysis on Sciex TripleTOF6600 mass spectrometer interfaced with an Eksigent nano-LC 425 system using established protocols previously reported by us [92, 93]. All raw data was analyzed using the ProteinPilot software from Sciex using search parameters previously described by us [93]. The GO term for the differential peptides were plotted using g:Profiler. The colour of the circles indicate the specific dataset the terms belong to on the × axis, for example, Red = Molecular Function, Orange = Biological pathway, REAC = Reactome database, TF = Transcription Factor. The size of the circle indicates the number of genes correlated to that term as indicated above. The highlighted numbered circles have been selected to focus on the major terms that get significantly altered and have been mentioned in the list given in the Supplementary Tables 1–3. The mass spectrometry proteomics data have been deposited to the ProteomeXchange Consortium via the PRIDE [1] partner repository with the dataset identifier PXD041210.

### Immunofluorescence assay

HCT15 cells (1 × 10^4^) were seeded onto the 8 well chambered slides. After 3 days, when spheroids were formed then it was treated with statin (10 μM). After 48 h of treatment, cells were fixed with 4% paraformaldehyde (PFA) for 10 min followed by permeabilization with 0.1% Triton-X-100 for 10 mins. The cells were then blocked with 2% BSA for 1 h. Primary antibody (1:100 dilution) incubation was done overnight at 4°C followed by three 1X PBS washes for 10 mins each. The chambered slides were then incubated with secondary antibodies (1:200 dilution) for 1 h at room temperature followed by three 1X PBS washes for 10 mins each. The cells were then stained with DAPI for 10 mins followed by two 1X PBS washes for 10 mins each. The chambered slides were then imaged using a Nikon A1R confocal microscope. Antibodies used were as follows: SATB1 (SC-376096), SATB2 (ab92447), Vimentin (ab92547), E-cadherin (CST#14472).

### *In vivo* study of tumor in mice

HCT116 cells and SW480-FLAG overexpressing SATB1 cells, SW480-FLAG control cells (1 × 10^6^ each) were separately injected subcutaneously in 8 NOD-SCID mice (male or female respectively, 6–8 weeks old, weighing 25–30 g). They were equally divided into two sets, where one set was designated vehicle control, and the other set was given statin treatment. Simvastatin (given to mice injected with HCT116 cells) /rosuvastatin (given to mice injected with SW480 SATB1 overexpressing cells) were dissolved in 0.5% methylcellulose and administered orally daily for 45 days, post-7 days of injection of cells. The dose provided was 40 mg/kg/day and no side effects were observed. The mice were sacrificed after 45 days of the assay and the tumor was isolated and weighed. All the murine experiments were approved by the institutional animal ethics committee of IISER Pune and were conducted in house at the National Facility for Gene Function in Health and Disease.

### Statistical analysis

All experiments were performed in biological triplicates unless specified otherwise. Statistical analyses between two groups were performed using the two-tailed unpaired Student’s *t*-test. A confidence level of 0.05 was considered statistically significant unless stated otherwise.

## SUPPLEMENTARY MATERIALS


