# Supplementary material for: Statins exhibit anti-tumor potential by modulating Wnt/β-catenin signaling in colorectal cancer
Source: Oncotarget. 2025 Jul 21;16:562–81. doi: 10.18632/oncotarget.28755 (PMC12279029; doi:10.18632/oncotarget.28755)
Supplement: Supplementary file 1 [file oncotarget-16-28755-s001.pdf]

Statins exhibit anti-tumor potential by modulating Wnt/β-catenin signaling in colorectal cancer

SUPPLEMENTARY MATERIALS

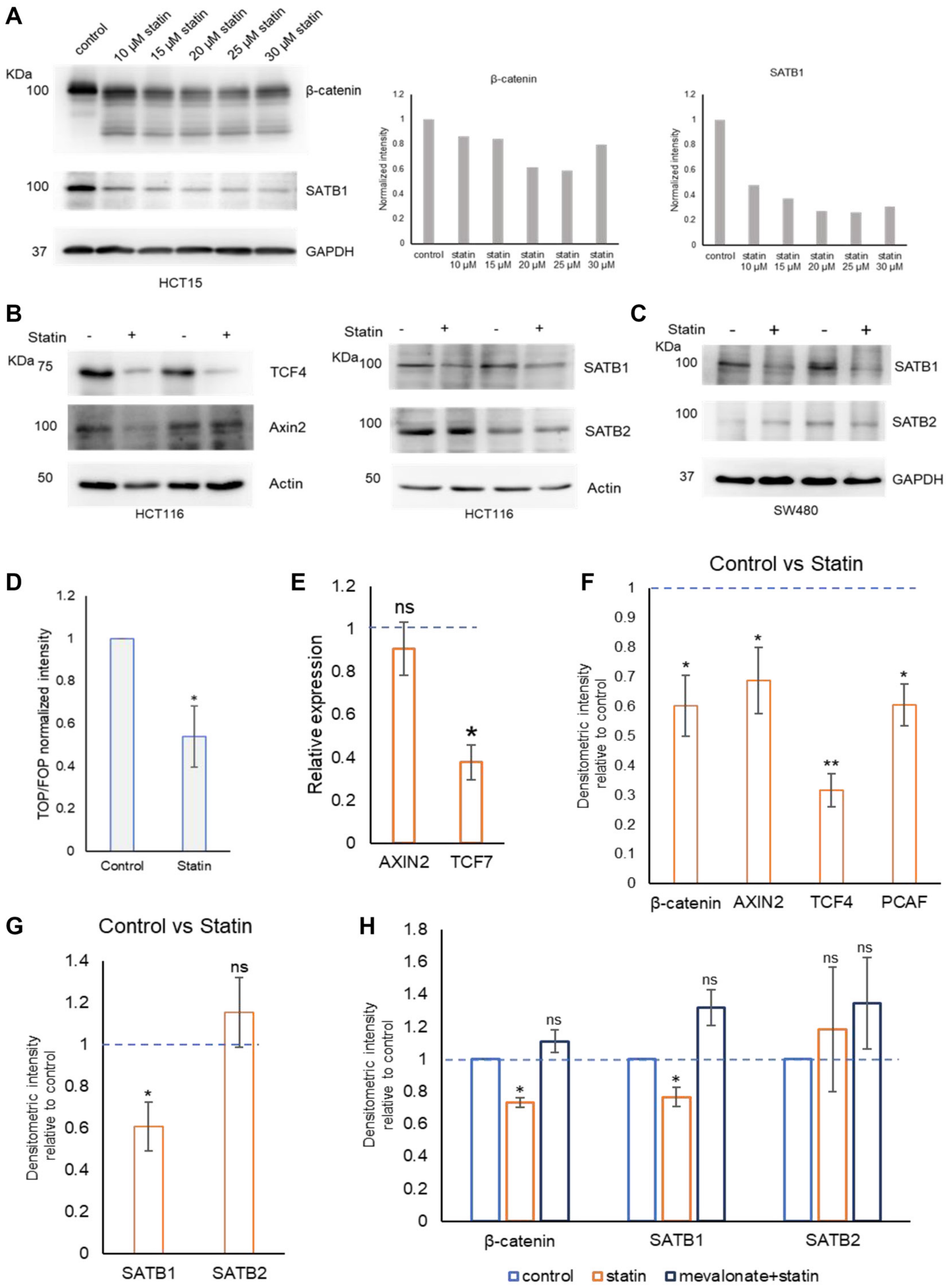

Supplementary Figure 1: Relative expression profiling of Wnt pathway players in simvastatin-treated cells as compared to control. (**A**) Immunoblot analysis was performed to assess the dose-dependent effects of simvastatin on β-catenin and SATB1 protein

levels in HCT15 cells. Densitometric quantification of band intensities, normalized and represented in the accompanying graphs, showed a dose-dependent reduction in both Wnt pathway components, with the most pronounced effect at 25 µM. However, to minimize cellular toxicity, we selected a 10 µM dose for subsequent experiments, as it produced a significant effect with minimal cell death. (**B**) Immunoblots were also conducted in HCT116 cells to evaluate the effect of simvastatin on various Wnt signaling components. We observed a reduction in TCF4 and AXIN2 levels, along with a modest decrease in SATB1. In contrast, SATB2 levels remained largely unchanged, consistent with the results in HCT15 cells. (**C**) A similar immunoblot experiment in SW480 cells demonstrated a reduction in SATB1 expression following simvastatin treatment, supporting the drug's efficacy across multiple colorectal cancer (CRC) cell lines. (**D**) A TOP/FOP flash luciferase assay was performed in HCT116 cells to assess the specific impact of simvastatin on Wnt/β-catenin signaling. Bioluminescence intensity was measured in cells transfected with the TOP construct, with and without simvastatin treatment, and normalized against the FOP negative control. A significant decrease in luminescence was observed in the simvastatin-treated group, indicating that the Wnt pathway is a specific target of the drug. (**E**) Quantitative PCR confirmed the downregulation of Wnt-responsive genes following simvastatin treatment. The graph illustrates the relative mRNA expression levels of AXIN2 and TCF7 in control versus statin-treated cells, showing a significant reduction in TCF7 expression. These findings are consistent with the transcriptomic analysis presented in Figure 2C. (**F**) Densitometric intensity relative to control for immunoblots of β-catenin, AXIN2, TCF4 and PCAF depicted in Figure 3B. All proteins exhibited a significant reduction upon simvastatin treatment. (**G**) Densitometric intensity relative to control for immunoblots of SATB1 and SATB2 in Figure 3D. SATB1 is observed to be significantly downregulated, whereas SATB2 did not exhibit a significant alteration. (**H**) Densitometric analysis relative to control for immunoblots of β-catenin, SATB1 and SATB2 in Figure 4A. Simvastatin treatment resulted in a reduction in protein levels of β-catenin and SATB1, whereas mevalonate plus statin treated cells exhibit a rescue. However, SATB2 levels were not significantly altered in both statin and mevalonate plus statin treated cells (Biological replicates $n = 3$, $^{*}p < 0.05$, $^{**}p < 0.005$, ns stands for non-significant upon Students' *t*-test analysis).

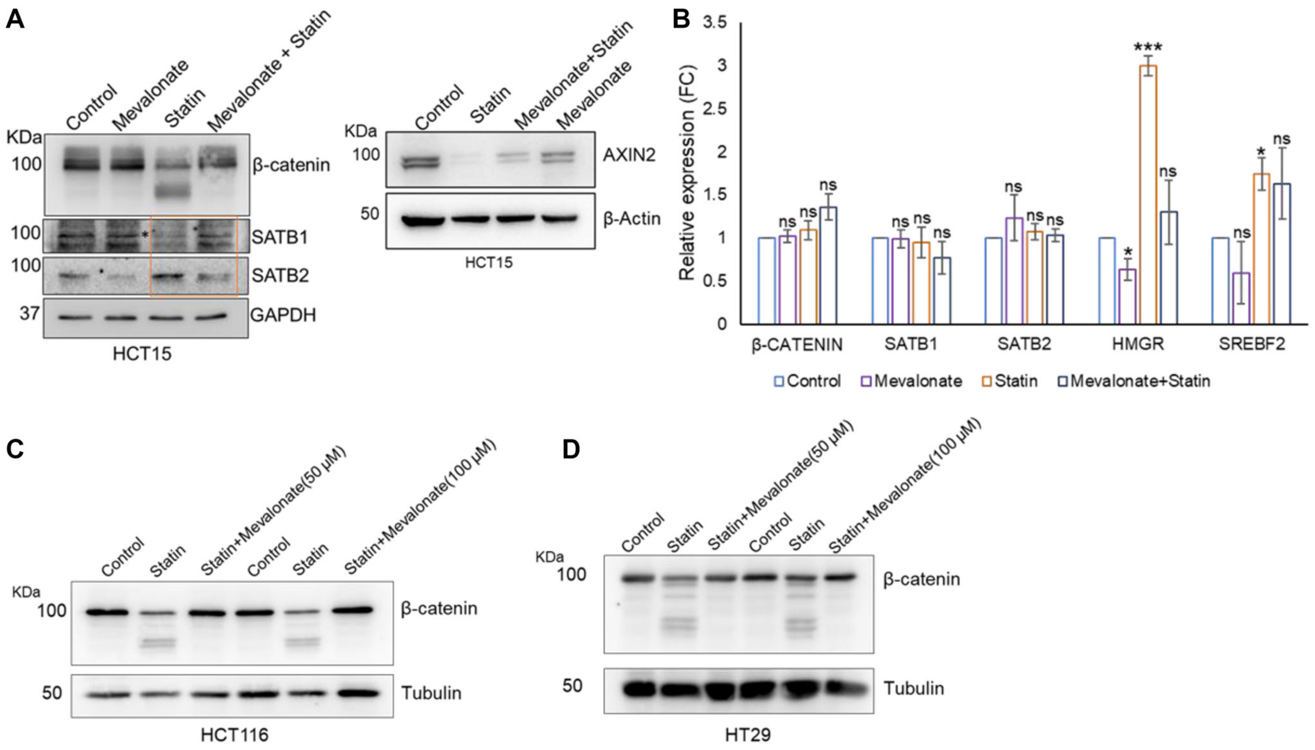

Supplementary Figure 2: Mevalonate supplementation along with simvastatin treatment rescues the protein levels of SATB1. (**A**) Immunoblot analysis of β-catenin, SATB1, SATB2, and AXIN2 protein levels under mevalonate, mevalonate + statin, and control treatment conditions. The upper band (* outside the red box) in the SATB1 blot corresponds to its expected molecular weight on SDS-PAGE. The statin-induced reduction in SATB1 levels was reversed upon mevalonate supplementation. SATB2 levels remained comparable to untreated controls following mevalonate treatment. Although simvastatin led to a modest increase in SATB2 expression, this was also restored to baseline with mevalonate. β-catenin and AXIN2 showed substantial reduction upon statin treatment, which was similarly rescued by mevalonate addition. (**B**) Relative gene expression profiling of β-catenin, SATB1, SATB2, HMGR and SREBF2 respectively under statin, mevalonate and mevalonate + statin conditions. No significant changes were detected in β-catenin, SATB1, or SATB2 transcript levels. However, the cholesterol-responsive genes HMGR and SREBF2 were upregulated upon statin treatment, with their expression levels returning to baseline upon co-treatment with mevalonate. (**C**, **D**) Immunoblot analysis was performed to assess the effect of simvastatin on β-catenin levels in HCT116 and HT29 cell lines. A marked reduction in β-catenin protein levels was observed in both cell lines following simvastatin treatment, along with the appearance of degradation products. This effect was reversed upon supplementation with mevalonate, mirroring the findings previously observed in HCT15 cells. (Biological replicates $n = 3$, $^{*}p < 0.05$, $^{***}p < 0.0005$, ns stands for non-significant by Students' *t*-test analysis).

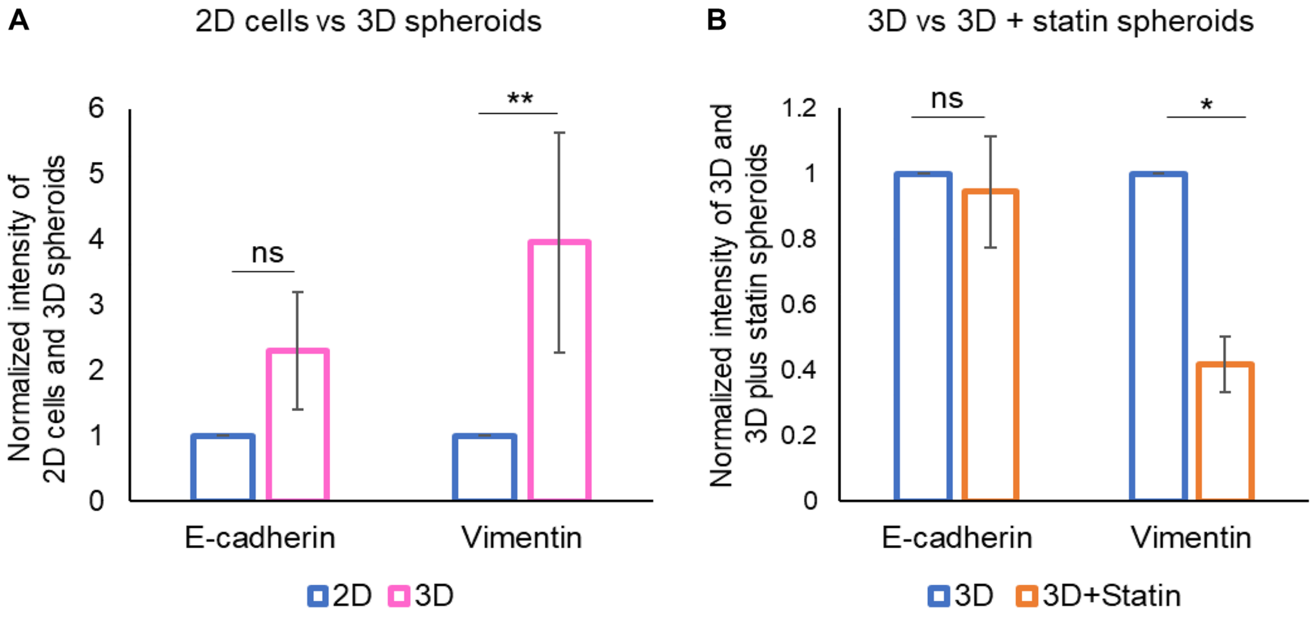

Supplementary Figure 3: Analysis of EMT markers in 2D cells versus 3D spheroids and vehicle control treated spheroids versus simvastatin-treated spheroids. (**A**) Densitometric analysis of immunoblot intensities for E-cadherin and Vimentin in 3D spheroids relative to 2D cultured cells (as shown in Figure 5C). While E-cadherin expression remained largely unchanged between 2D and 3D conditions, Vimentin levels were significantly elevated in the spheroids, indicating the induction of an EMT phenotype. (**B**) Densitometric analysis of normalized immunoblot intensities for E-cadherin and Vimentin in spheroids following simvastatin treatment (Figure 6D). Simvastatin treatment led to a marked reduction in Vimentin expression, suggesting a partial reversal of the EMT phenotype. However, E-cadherin levels were not significantly altered. (Biological replicates $n = 3$, $^{*}p < 0.05$, $^{**}p < 0.005$, ns stands for non-significant upon Students' *t*-test analysis).

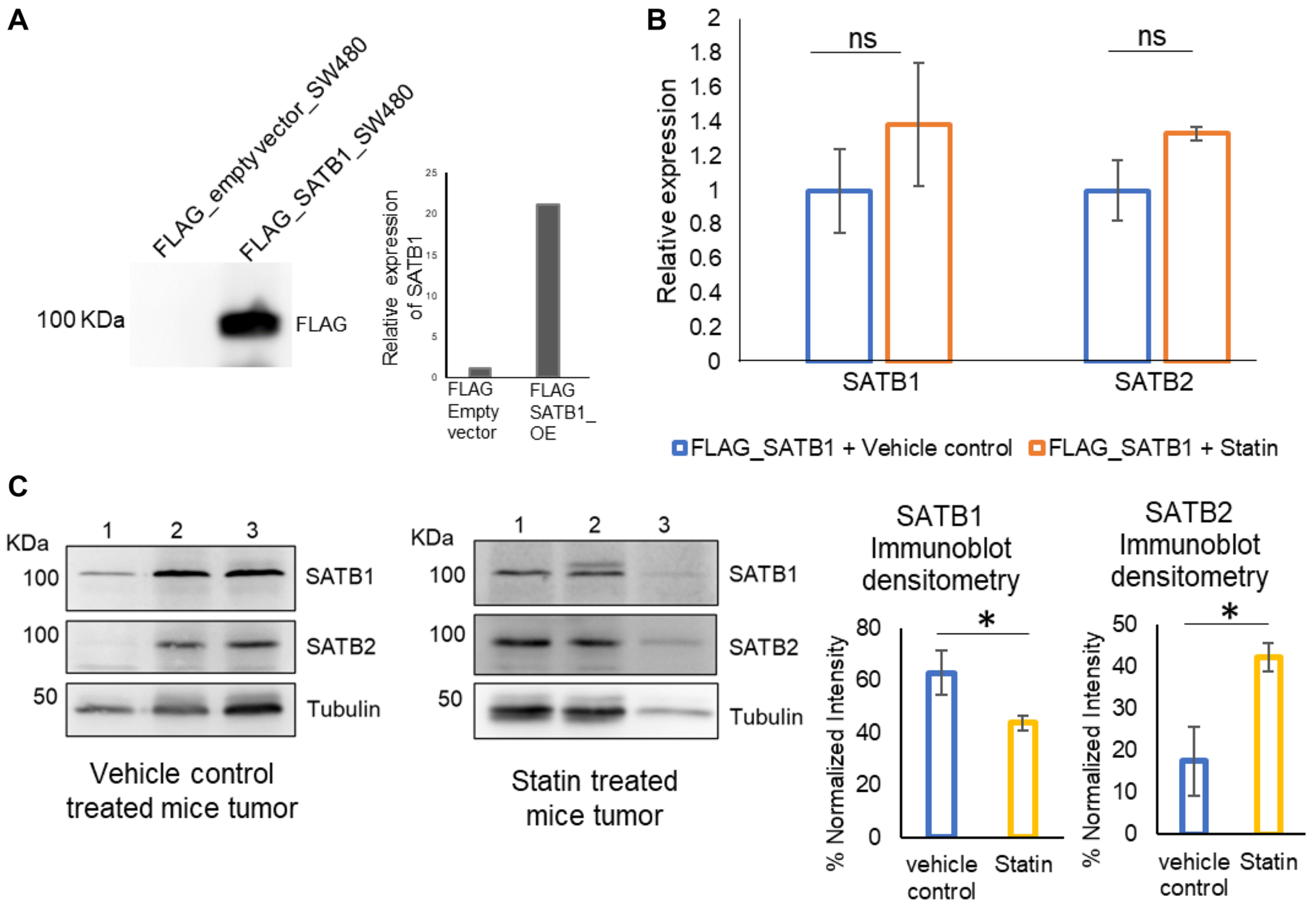

Supplementary Figure 4: Molecular profiling of the tumor tissues isolated from rosuvastatin treated NOD-SCID mice suggests downregulation of SATB1. (**A**) Validation of FLAG-SATB1 over-expression in the SW480 cell line implanted in mice, assessed at both the protein and transcript levels. (**B**) Transcript levels of SATB1 and SATB2 in tumor tissues from mice treated with either vehicle control or rosuvastatin show no significant changes. (**C**) Immunoblots displaying SATB1 and SATB2 protein levels in tumors from both vehicle control and rosuvastatin-treated mice. SATB1 levels appear reduced in the rosuvastatin treatment group, supporting the in-cell data. The densitometry analysis graph on the right indicates a significant downregulation of SATB1 in tumor samples from rosuvastatin-treated mice. (Biological replicates $n = 3$, $^*p < 0.05$ and ns stands for non-significant by Students' *T*-test).

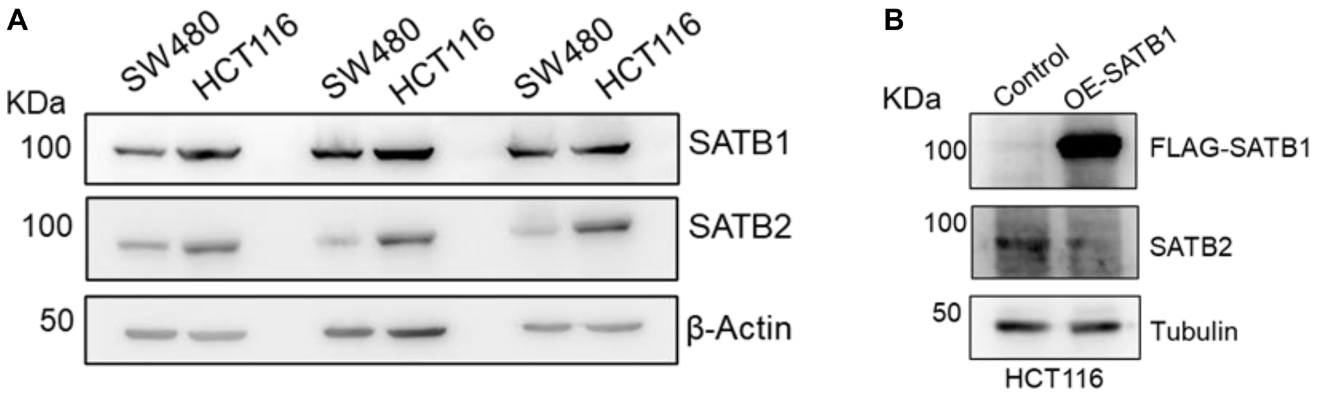

Supplementary Figure 5: Signature reciprocal expression profile of SATB proteins in CRC cell lines. (**A**) Endogenous SATB1 and SATB2 protein levels in SW480 and HCT116 cell lines reveal that SATB1 is more highly expressed in the more aggressive HCT116 cells compared to SW480. In both lines, SATB2 expression is lower than SATB1, indicating a reciprocal relationship between the two homologs. (**B**) Ectopic overexpression of SATB1 in HCT116 cells further reinforces this inverse relationship, with increased SATB1 levels leading to a marked reduction in SATB2 protein expression.

Supplementary Table 1: GO terms list for the Manhattan plot (Figure 2A) provided from g:Profiler software for the significantly upregulated genes on simvastatin treatment in transcriptome analysis

ID	Source	Term ID	Term Name	p_{adj} (query_1)
1	GO:BP	GO:0001676	long-chain fatty acid metabolic process	2.214×10^{-3}
2	GO:BP	GO:0006082	organic acid metabolic process	1.894×10^{-4}
3	GO:BP	GO:0006631	fatty acid metabolic process	1.147×10^{-4}
4	GO:BP	GO:0008299	isoprenoid biosynthetic process	5.599×10^{-5}
5	GO:BP	GO:0010033	response to organic substance	1.691×10^{-5}
6	GO:BP	GO:0019216	regulation of lipid metabolic process	9.291×10^{-8}
7	GO:BP	GO:0006629	lipid metabolic process	1.334×10^{-8}
8	GO:BP	GO:0008610	lipid biosynthetic process	1.505×10^{-9}
9	GO:BP	GO:0019218	regulation of steroid metabolic process	1.208×10^{-9}
10	GO:BP	GO:0006066	alcohol metabolic process	3.867×10^{-13}
11	GO:BP	GO:0006694	steroid biosynthetic process	6.240×10^{-14}
12	GO:BP	GO:0008202	steroid metabolic process	1.721×10^{-15}
13	GO:BP	GO:0008203	cholesterol metabolic process	2.319×10^{-17}
14	GO:BP	GO:0006695	cholesterol biosynthetic process	1.378×10^{-19}
15	GO:BP	GO:0016125	sterol metabolic process	1.678×10^{-19}
16	GO:BP	GO:0045540	regulation of cholesterol biosynthetic process	4.569×10^{-13}
17	GO:BP	GO:0046165	alcohol biosynthetic process	1.549×10^{-12}
18	GO:BP	GO:0090181	regulation of cholesterol metabolic process	6.932×10^{-12}
19	GO:BP	GO:0106118	regulation of sterol biosynthetic process	4.569×10^{-13}
20	GO:BP	GO:1902652	secondary alcohol metabolic process	4.145×10^{-17}
21	GO:BP	GO:1901617	organic hydroxy compound biosynthetic process	2.775×10^{-13}
22	GO:BP	GO:0046890	regulation of lipid biosynthetic process	7.293×10^{-10}
23	GO:BP	GO:0050810	regulation of steroid biosynthetic process	9.586×10^{-11}
24	GO:BP	GO:0044283	small molecule biosynthetic process	1.603×10^{-9}
25	GO:BP	GO:0044281	small molecule metabolic process	2.021×10^{-8}
26	GO:BP	GO:0044255	cellular lipid metabolic process	1.758×10^{-5}
27	GO:BP	GO:0033993	response to lipid	4.828×10^{-4}
28	GO:BP	GO:0019752	carboxylic acid metabolic process	3.530×10^{-5}
29	GO:BP	GO:0046949	fatty-acyl-CoA biosynthetic process	1.644×10^{-4}
30	GO:BP	GO:0071616	acyl-CoA biosynthetic process	5.229×10^{-4}
31	GO:BP	GO:0006637	acyl-CoA metabolic process	6.871×10^{-3}
32	GO:BP	GO:1901568	fatty acid derivative metabolic process	1.351×10^{-5}
33	KEGG	KEGG:01212	Fatty acid metabolism	1.176×10^{-2}
34	KEGG	KEGG:01100	Metabolic pathways	1.081×10^{-5}
35	REAC	REAC:R-HSA-89...	Fatty acid metabolism	3.210×10^{-3}
36	REAC	REAC:R-HSA-68...	Cholesterol biosynthesis via lathosterol	8.372×10^{-4}
37	REAC	REAC:R-HSA-75...	Fatty acyl-CoA biosynthesis	2.887×10^{-4}
38	REAC	REAC:R-HSA-14...	Metabolism	2.976×10^{-4}
39	WP	WP:WP4804	Cholesterol Biosynthesis with Skeletal Dysplasias	7.289×10^{-6}
40	WP	WP:WP3963	Mevalonate pathway	7.289×10^{-6}
41	WP	WP:WP1982	Sterol Regulatory Element-Binding Proteins (SREB...	6.915×10^{-7}
42	REAC	REAC:R-HSA-55...	Metabolism of lipids	4.046×10^{-9}
43	REAC	REAC:R-HSA-16...	Regulation of cholesterol biosynthesis by SREBP (S...	1.142×10^{-10}
44	REAC	REAC:R-HSA-89...	Metabolism of steroids	7.705×10^{-12}
45	REAC	REAC:R-HSA-24...	Activation of gene expression by SREBF (SREBP)	6.627×10^{-12}
46	WP	WP:WP197	Cholesterol Biosynthesis Pathway	1.815×10^{-16}
47	WP	WP:WP4718	Cholesterol metabolism (includes both Bloch and ...	8.528×10^{-22}
48	REAC	REAC:R-HSA-19...	Cholesterol biosynthesis	7.511×10^{-21}
49	KEGG	KEGG:00100	Steroid biosynthesis	1.343×10^{-13}
50	WP	WP:WP2011	SREBF and miR33 in cholesterol and lipid homeost...	3.801×10^{-4}
51	WP	WP:WP357	Fatty Acid Biosynthesis	4.500×10^{-3}
52	WP	WP:WP4724	Omega-9 FA synthesis	2.399×10^{-3}
53	REAC	REAC:R-HSA-45...	Interleukin-2 family signaling	1.245×10^{-2}
54	MIRNA	MIRNA:hsa-miR...	hsa-miR-335-5p	9.831×10^{-14}
55	TF	TF:M10108	Factor: WT1; motif: RGGNGGGGGAGGRGGNGGRG	1.687×10^{-2}
56	TF	TF:M12160	Factor: KLF15; motif: RCCMCRCCCMCN	4.480×10^{-2}
57	TF	TF:M07289	Factor: GKLF; motif: NNNRGGNGNGGSN	4.670×10^{-4}
58	TF	TF:M07040	Factor: GKLF; motif: NNRRGRRNGNSNNN	2.406×10^{-4}
59	TF	TF:M00915_0	Factor: AP-2; motif: SNNNCCNCAGGCN; match cla...	3.099×10^{-3}
60	TF	TF:M00444	Factor: VDR; motif: GGGKNARNRRGGWSA	9.565×10^{-3}

The color coding is dependent on the significance value (*p*-value) assigned to the terms in the software based on the gene list belonging to the term and the statistical significance of their match, blue signifying highly significant to orange signifying least significant. Therefore, all the GO terms listed are highly significant.

Supplementary Table 2: GO terms list for the Manhattan plot (Figure 2B) provided from g:Profiler software for the significantly downregulated genes on simvastatin treatment in transcriptome analysis

ID	Source	Term ID	Term Name	p_{adj} (query_1)
1	GO:MF	GO:0003677	DNA binding	3.816×10^{-2}
2	GO:MF	GO:0003824	catalytic activity	1.268×10^{-2}
3	GO:MF	GO:0000217	DNA secondary structure binding	3.312×10^{-3}
4	GO:MF	GO:0003676	nucleic acid binding	2.715×10^{-6}
5	GO:MF	GO:0033170	protein-DNA loading ATPase activity	1.165×10^{-3}
6	GO:MF	GO:0003697	single-stranded DNA binding	4.434×10^{-8}
7	GO:BP	GO:0007049	cell cycle	1.115×10^{-22}
8	GO:BP	GO:0022402	cell cycle process	2.890×10^{-20}
9	REAC	REAC:R-HSA-16...	Cell Cycle	2.733×10^{-23}
10	REAC	REAC:R-HSA-69...	Cell Cycle, Mitotic	3.724×10^{-18}
11	WP	WP:WP2363	Gastric Cancer Network 2	6.292×10^{-4}
12	WP	WP:WP2446	Retinoblastoma Gene in Cancer	2.425×10^{-13}
13	WP	WP:WP179	Cell Cycle	3.092×10^{-9}
14	TF	TF:M04826_0	Factor: p300; motif: ACNTCCG; match class: 0	2.934×10^{-2}
15	TF	TF:M03867_0	Factor: c-Myc; motif: CACGTGGC; match class: 0	2.126×10^{-2}
16	TF	TF:M01145	Factor: c-Myc; motif: RACCACGTGCTC	5.714×10^{-5}
17	TF	TF:M07601_1	Factor: C-Myc; motif: NGCCACGTGNN; match class...	2.719×10^{-5}
18	TF	TF:M01154	Factor: c-Myc; motif: KACCACGTGSYY	9.438×10^{-7}
19	TF	TF:M01145_1	Factor: c-Myc; motif: RACCACGTGCTC; match class:...	3.938×10^{-7}
20	TF	TF:M07206_1	Factor: E2F-1; motif: NGGGCGGGARV; match class: 1	2.744×10^{-8}
21	TF	TF:M07601_0	Factor: C-Myc; motif: NGCCACGTGNN; match class...	5.262×10^{-9}
22	TF	TF:M11531_1	Factor: E2F-2; motif: GCGCGCGCGYW; match class: 1	5.023×10^{-10}
23	TF	TF:M04743	Factor: c-Myc; motif: NSCACGTGGN	1.897×10^{-11}
24	TF	TF:M00797_0	Factor: HIF1; motif: GNNKACGTGCGGNN; match cl...	3.022×10^{-4}
25	TF	TF:M07384_0	Factor: HIF-1alpha; motif: NCACGTNN; match class...	1.318×10^{-2}
26	TF	TF:M00322_1	Factor: c-Myc:Max; motif: GCCAYGYGSN; match cla...	6.492×10^{-3}
27	TF	TF:M09992_1	Factor: c-Myc; motif: NCCACGTGCNN; match class: 1	1.086×10^{-3}
28	TF	TF:M11081_1	Factor: SREBP-1; motif: RTCRCGTGAY; match class: 1	2.337×10^{-8}
29	TF	TF:M11081	Factor: SREBP-1; motif: RTCRCGTGAY	1.624×10^{-5}
30	TF	TF:M11081_0	Factor: SREBP-1; motif: RTCRCGTGAY; match class: 0	1.624×10^{-5}
31	TF	TF:M11601	Factor: TCF-1; motif: ACATCGRGRCGCTGW	3.351×10^{-4}
32	TF	TF:M11603_1	Factor: TCF-1; motif: ACATCGRGRCGCTGW; match ...	1.993×10^{-2}
33	TF	TF:M11603	Factor: TCF-1; motif: ACATCGRGRCGCTGW	2.831×10^{-2}
34	MIRNA	MIRNA:hsa-miR...	hsa-miR-215-5p	1.790×10^{-11}
35	MIRNA	MIRNA:hsa-miR...	hsa-miR-192-5p	5.363×10^{-12}
36	MIRNA	MIRNA:hsa-miR...	hsa-miR-193b-3p	2.609×10^{-15}
37	GO:CC	GO:0005657	replication fork	6.730×10^{-9}

The color coding is dependent on the significance value (*p*-value) assigned to the terms in the software based on the gene list belonging to the term and the statistical significance of their match, blue signifying highly significant to orange signifying least significant. Therefore, all the GO terms listed are highly significant.

Supplementary Table 3: GO terms list for the Manhattan plot (Figure 3A) provided from g:Profiler software for the significantly downregulated proteins on simvastatin treatment in whole lysate proteomics MS-MS analysis

ID	Source	Term ID	Term Name	p_{adj} (query_1)
1	GO:MF	GO:0045296	cadherin binding	9.638×10^{-12}
2	GO:MF	GO:0050839	cell adhesion molecule binding	1.710×10^{-8}
3	GO:MF	GO:0097159	organic cyclic compound binding	2.638×10^{-4}
4	GO:MF	GO:1901363	heterocyclic compound binding	3.704×10^{-4}
5	GO:MF	GO:0031625	ubiquitin protein ligase binding	2.489×10^{-2}
6	GO:BP	GO:1901575	organic substance catabolic process	1.332×10^{-10}
7	GO:BP	GO:0043161	proteasome-mediated ubiquitin-dependent protei...	1.756×10^{-5}
8	GO:BP	GO:0010498	proteasomal protein catabolic process	1.065×10^{-4}
9	GO:BP	GO:0051603	proteolysis involved in cellular protein catabolic pr...	4.716×10^{-4}
10	GO:BP	GO:0006511	ubiquitin-dependent protein catabolic process	5.869×10^{-3}
11	GO:BP	GO:0017015	regulation of transforming growth factor beta rece...	6.255×10^{-3}
12	GO:BP	GO:1903844	regulation of cellular response to transforming gr...	7.426×10^{-3}
13	GO:BP	GO:0070498	interleukin-1-mediated signaling pathway	1.620×10^{-2}
14	GO:BP	GO:0016055	Wnt signaling pathway	3.712×10^{-2}
15	GO:BP	GO:0198738	cell-cell signaling by wnt	3.870×10^{-2}
16	REAC	REAC:R-HSA-21...	Downregulation of TGF-beta receptor signaling	6.832×10^{-7}
17	REAC	REAC:R-HSA-89...	Regulation of PTEN localization	3.356×10^{-6}
18	REAC	REAC:R-HSA-21...	TGF-beta receptor signaling activates SMADs	3.834×10^{-6}
19	REAC	REAC:R-HSA-46...	Degradation of DVL	2.260×10^{-5}
20	REAC	REAC:R-HSA-17...	Signaling by TGF-beta Receptor Complex	1.304×10^{-4}
21	REAC	REAC:R-HSA-21...	Regulation of activated PAK-2p34 by proteasome ...	1.609×10^{-4}
22	REAC	REAC:R-HSA-12...	Downregulation of ERBB4 signaling	2.027×10^{-4}
23	REAC	REAC:R-HSA-34...	Autodegradation of the E3 ubiquitin ligase COP1	2.121×10^{-4}
24	REAC	REAC:R-HSA-75...	Ubiquitin-dependent degradation of Cyclin D	2.121×10^{-4}
25	REAC	REAC:R-HSA-69...	Ubiquitin Mediated Degradation of Phosphorylate...	2.121×10^{-4}
26	REAC	REAC:R-HSA-89...	Regulation of RUNX3 expression and activity	2.764×10^{-4}
27	REAC	REAC:R-HSA-46...	Degradation of AXIN	3.143×10^{-4}
28	REAC	REAC:R-HSA-17...	SCF-beta-TrCP mediated degradation of Emi1	3.143×10^{-4}
29	REAC	REAC:R-HSA-88...	PTK6 Regulates RTKs and Their Effectors AKT1 and...	3.623×10^{-4}
30	REAC	REAC:R-HSA-90...	Signaling by NOTCH4	3.643×10^{-4}
31	REAC	REAC:R-HSA-56...	NIK-->noncanonical NF-kB signaling	5.121×10^{-4}
32	REAC	REAC:R-HSA-56...	Hedgehog 'on' state	5.281×10^{-4}
33	REAC	REAC:R-HSA-18...	SCF(Skp2)-mediated degradation of p27/p21	5.752×10^{-4}
34	REAC	REAC:R-HSA-56...	GLI3 is processed to GLI3R by the proteasome	5.752×10^{-4}
35	REAC	REAC:R-HSA-56...	Degradation of GLI2 by the proteasome	5.752×10^{-4}
36	REAC	REAC:R-HSA-56...	Degradation of GLI1 by the proteasome	5.752×10^{-4}
37	REAC	REAC:R-HSA-93...	IRAK2 mediated activation of TAK1 complex	5.993×10^{-4}
38	REAC	REAC:R-HSA-56...	Dectin-1 mediated noncanonical NF-kB signaling	5.752×10^{-4}
39	REAC	REAC:R-HSA-90...	TICAM1,TRAF6-dependent induction of TAK1 com...	9.349×10^{-4}
40	REAC	REAC:R-HSA-53...	Hedgehog ligand biogenesis	9.971×10^{-4}
41	REAC	REAC:R-HSA-17...	APC/C:Cdc20 mediated degradation of Securin	1.356×10^{-3}
42	REAC	REAC:R-HSA-13...	Downregulation of ERBB2:ERBB3 signaling	1.392×10^{-3}
43	REAC	REAC:R-HSA-90...	TICAM1-dependent activation of IRF3/IRF7	1.392×10^{-3}
44	REAC	REAC:R-HSA-90...	Signaling by TGFB family members	1.815×10^{-3}
45	REAC	REAC:R-HSA-17...	Cdc20:Phospho-APC/C mediated degradation of C...	2.192×10^{-3}
46	REAC	REAC:R-HSA-17...	APC/C:Cdh1 mediated degradation of Cdc20 and ...	2.402×10^{-3}
47	REAC	REAC:R-HSA-18...	EGFR downregulation	3.235×10^{-3}
48	REAC	REAC:R-HSA-97...	IRAK1 recruits IKK complex upon TLR7/8 or 9 stim...	2.774×10^{-3}
49	WP	WP:WP183	Proteasome Degradation	4.742×10^{-2}
50	TF	TF:M04515_1	Factor: E2F-1; motif: WWTGGCGCCAAA; match cla...	1.641×10^{-5}
51	TF	TF:M04826_1	Factor: p300; motif: ACNTCCG; match class: 1	1.561×10^{-4}
52	TF	TF:M11530	Factor: E2F-2; motif: NWTTTGGCGCCAWWNN	1.912×10^{-4}
53	TF	TF:M10438	Factor: ZF5; motif: GGSGCGCGS	5.816×10^{-4}
54	TF	TF:M00932_1	Factor: Sp1; motif: NNGGGGCGGGGNN; match cla...	8.032×10^{-4}
55	TF	TF:M12160	Factor: KLF15; motif: RCCMCRCCCMCN	1.247×10^{-2}
56	TF	TF:M03925	Factor: YY2; motif: NCCGCCATNTY	3.091×10^{-2}
57	TF	TF:M03924_1	Factor: YY1; motif: NNCGCCATTNN; match class: 1	4.267×10^{-2}
58	TF	TF:M10530	Factor: sp4; motif: NNNGCYCCGCCCCCY	3.445×10^{-2}
59	MIRNA	MIRNA:hsa-miR...	hsa-miR-615-3p	5.288×10^{-11}
60	MIRNA	MIRNA:hsa-miR...	hsa-miR-324-3p	2.115×10^{-2}

The color coding is dependent on the significance value (*p*-value) assigned to the terms in the software based on the protein list belonging to the term and the statistical significance of their match, blue signifying highly significant to orange signifying least significant. Therefore, all the GO terms listed are highly significant.

Supplementary Table 4: The list of qPCR primers used for the analysis

S.No.	Gene name	qPCR Primer sequence
1	SATB1	Forward- AACTCAGGGCTTGCTTTC<br>Reverse- CCTGGTATATTCGGTCTCTTTC
2	SATB2	Forward- AGAGATGAACCAGAGCACATTAG<br>Reverse- GTTGCTGACACATTGGCATAAT
3	β-catenin	Forward- AAGGTGTGGCGACATATGCA<br>Reverse- GTAATCTTGTGGCTTGTCCTCAGA
4	18s rRNA	Forward- CGCCGCTAGAGGTGAAATTCT<br>Reverse- CGAACCTCCGACTTTCGTTCT
5	SREBF2	Forward- GCTGCCAAGGAGAGTCTAT<br>Reverse- AGGTTTCACCAAGGACTCTAT
6	HMGR	Forward- ACAAGAATTTAGTGGGCTCTG<br>Reverse- TCCTGTCCACAGGCAAT
7	CDH1	Forward- GGCTGGACCGAGAGAGTTTC<br>Reverse- CCTGACCCTTGTACGTGGTG
8	Vimentin	Forward- GCGAGGAGAGCAGGATTTCT<br>Reverse- TGGGTATCAACCAGAGGGAGT
9	E-cadherin	Forward- GTCCTGGGCAGAGTGAAT<br>Reverse- GGGTTATGAAACCGTAGAGGC
